# The structure of songbird MHC class I reveals antigen binding that is flexible at the N-terminus and static at the C-terminus

**DOI:** 10.3389/fimmu.2023.1209059

**Published:** 2023-07-07

**Authors:** Sandra Eltschkner, Samantha Mellinger, Soren Buus, Morten Nielsen, Kajsa M. Paulsson, Karin Lindkvist-Petersson, Helena Westerdahl

**Affiliations:** ^1^ Molecular Plant Pathology, Swammerdam Institute for Life Sciences, University of Amsterdam, Amsterdam, Netherlands; ^2^ Molecular Ecology and Evolution Lab, Department of Biology, Lund University, Lund, Sweden; ^3^ Department of Experimental Immunology, Institute of International Health, Immunology and Microbiology, Copenhagen, Denmark; ^4^ Immunoinformatics and Machine Learning, Department of Health Technology, Technical University of Denmark, Lyngby, Denmark; ^5^ Antigen Presentation, Department of Experimental Medical Science, Lund University, Lund, Sweden; ^6^ Medical Structural Biology, Department of Experimental Medical Science, Lund University, Lund, Sweden; ^7^ LINXS - Institute of Advanced Neutron and X-ray Science, Lund University, Lund, Sweden

**Keywords:** Major Histocompatibility Complex, MHC class I, Passeriformes, X-ray structure, antigen presentation, great reed warbler

## Abstract

Long-distance migratory animals such as birds and bats have evolved to withstand selection imposed by pathogens across the globe, and pathogen richness is known to be particularly high in tropical regions. Immune genes, so-called Major Histocompatibility Complex (MHC) genes, are highly duplicated in songbirds compared to other vertebrates, and this high MHC diversity has been hypothesised to result in a unique adaptive immunity. To understand the rationale behind the evolution of the high MHC genetic diversity in songbirds, we determined the structural properties of an MHC class I protein, Acar3, from a long-distance migratory songbird, the great reed warbler *Acrocephalus arundinaceus* (in short: *Acar*). The structure of Acar3 was studied in complex with pathogen-derived antigens and shows an overall antigen presentation similar to human MHC class I. However, the peptides bound to Acar3 display an unusual conformation: Whereas the N-terminal ends of the peptides display enhanced flexibility, the conformation of their C-terminal halves is rather static. This uncommon peptide-binding mode in Acar3 is facilitated by a central Arg residue within the peptide-binding groove that fixes the backbone of the peptide at its central position, and potentially permits successful interactions between MHC class I and innate immune receptors. Our study highlights the importance of investigating the immune system of wild animals, such as birds and bats, to uncover unique immune mechanisms which may neither exist in humans nor in model organisms.

## Introduction

Songbirds belong to the most species-rich bird order on earth, Passeriformes (1). They cross the globe during their annual migratory journeys and manage to breed in a wide range of different habitats (2–4). The successful radiation with regards to number of species suggests that songbirds have been very adaptable to different environments, and hence able to handle selection from a wide range of pathogens (5–7). However, these adaptations are not unique to songbirds, as all vertebrates in the tropics, where pathogens are particularly numerous, have evolved immune systems capable of withstanding a diverse spectrum of pathogens (8–10). In recent time, the immune system in bats, forming the second largest order among mammals, has been scrutinised in detail since many bats carry a multitude of viruses (Ebola, Nipah, severe acute respiratory syndrome (SARS) and Middle East respiratory syndrome (MERS)) seemingly without any symptoms, whereas humans become severely ill upon infection with these viruses (11–16). It is less known to what extent songbirds carry pathogens without showing symptoms yet bearing the risk of becoming zoonotic.

Interestingly, bats and songbirds display a similar evolutionary adaptation in their adaptive immune system, since their Major Histocompatibility Complex class I (MHC-I) genes have expanded more than in other terrestrial vertebrates, with many songbirds harbouring vastly duplicated MHC-I genes (> 50 alleles per individual) (17–20). MHC-I molecules are expressed on all nucleated cells and are central in adaptive immune responses towards intracellular pathogens, such as viruses and intracellular bacteria (21). MHC-I molecules present antigens to CD8^+^ T-cells, and if the antigen is pathogen-derived, the cell will be killed, whereas cells only displaying self-antigens are left untouched. Each MHC-I molecule can present a small fraction of antigens. The range of those antigens, which contain a preferred set of anchor residues, is determined by the properties of the peptide-binding groove (PBG).

Theoretically, a very high MHC diversity (number of different MHC alleles per individual) is unfavourable as MHC diversity higher than the optimal diversity will diminish the total T-cell repertoire (22). However, the strength of the negative selection of T-cells, *i.e.* central tolerance, is correlated not only with the MHC diversity *per se*, but also with the average antigen-binding spectrum of individual MHC-I molecules: a narrow repertoire allows a higher MHC diversity than a broader repertoire per MHC-I molecule (23, 24). Humans have six MHC-I genes, three classical and three non-classical, whereas in bats up to 12 MHC-I-like genes have been reported (17) which corresponds well with bats having a potentially narrower peptide-binding repertoire per individual MHC-I molecule than humans (25–27). Songbirds have at least 20 expressed MHC-I alleles (28), *i.e.* between 10 and 20 MHC-I genes depending on degree of heterozygosity, suggesting that songbird MHC-I molecules could have an even more restricted repertoire than humans and bats. Although the average antigen-binding breadth is expected to differ among species, MHC-I molecules with narrow (fastidious) and broad (promiscuous) antigen binding repertoires probably exist in most species, as shown in recent comparative work from humans and the avian model species, chicken (*Gallus gallus*), the latter with only two classical MHC-I genes (29).

Here we set out to determine the structural properties of Acar3, the most highly expressed MHC-I molecule from a long-distance migratory songbird, the great reed warbler *Acrocephalus arundinaceus* (*Acar*). In this species, that breeds in the Western Palearctic and winters in Sub-Saharan Africa (30), the MHC-I diversity has been thoroughly characterised (31–33). Our structural studies on the Acar3-heavy chain in complex with *Acar* beta-2-microglobulin (β_2_m) and two different peptides reveal a surprisingly high N-terminal malleability of those peptides, despite sharing a Met residue acting as N-terminal anchor. Intriguingly, an Arg residue at the centre of the PBG which binds to the backbone of the peptides leads to highly similar conformations within their C-terminal halves. This unique peptide conformation in Acar3 could have important implications for the immune competence of the great reed warbler, potentially enabling a more effective control of infection through counteracting pathogen escape mechanisms.

## Results

### The top-binding peptides of Acar3 show a preference for distinct anchor residues

**Figure 1:**
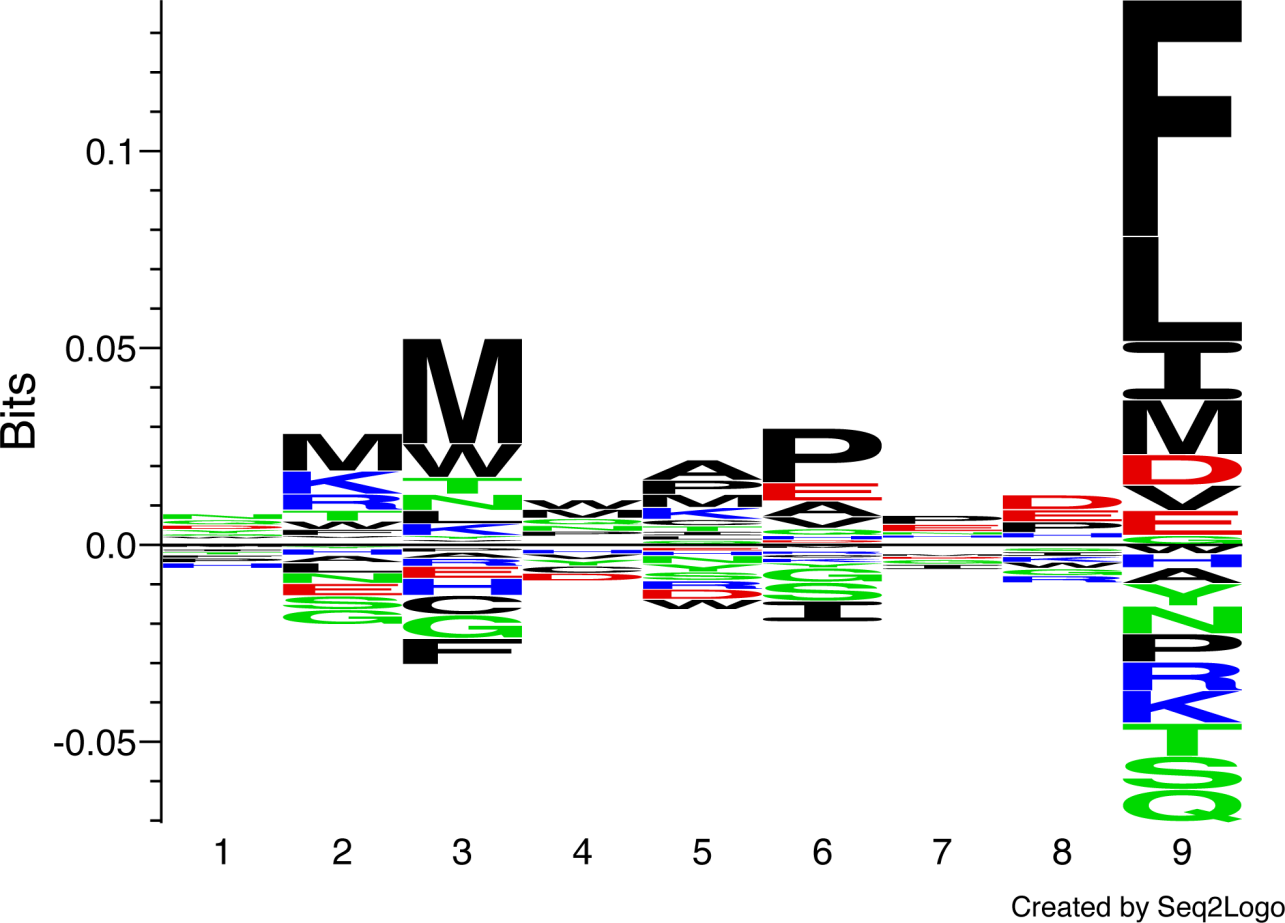
Peptide-binding logo for Acar3 based on PSCPL analysis using 9-mer peptide libraries. The peptide-binding logo was constructed with Seq2Logo 1.1 (35) based on the PSCPL-derived binding matrix (**Figure S2**). The relative height of each letter in the motif relates to the frequency of a given amino acid at that position in the bound 9-mer peptide.

MHC-I molecules bind short peptides of varying lengths, and after testing the length preferences of two different great reed warbler MHC-I molecules (**Figure S1**), we applied a 9-mer positional scanning combinatorial peptide library (PSCPL) approach to capture the peptide-binding motif and select peptides that can be presented by Acar3 (34). A PSCPL generated for nonameric peptides encompasses 20^9^ peptides (theoretically) when each position of the peptide is tested against all possible combinations of all other positions using all 20 natural amino acids. Subsequently, a homogenous Scintillation Proximity Assay (SPA), detecting the peptide-dependent binding of radiolabelled β_2_m to the Acar3 heavy chain, was used to monitor the stability of the peptide-MHC-I complexes generated. The relative contribution (relative binding (RB) value) of each sub-library (*i.e.* the compilation of peptides which reflects the position-specific effect of a defined amino acid at a specific position of the peptide comprising 1 of the 20 natural amino acids (including cysteine), whereas the other eight positions comprise an equimolar pool of 19 of the 20 natural amino acids (excluding cysteine)) to peptide binding was calculated (*see Materials and methods*) and the RB values of each amino acid in a given position were summarised and normalised so the sum equals 20 for each position. A matrix using the RB values was then generated where an RB value ≥ 2.0 defines a favoured amino acid at a specific position, and an RB value of ≤ 0.5 defines a disfavoured amino acid at a specific position (**Figure S2**). The PSCPL analysis reveals two dominant anchors in Acar3, at peptide positions three and nine, with a clear preference for hydrophobic residues. In particular Met (M) at position three and Phe (F) at position nine show the highest RB values of 2.1 and 2.6, respectively (**Figures 1**, **S2**). The anchor at position three of the peptide shows few disfavoured amino acids, *i.e.* only Phe (F) (RB = 0.3), whereas at position nine, there are four residues types, such as Lys (K, RB = 0.3), Gln (Q, RB = 0.4), Ser (S, RB = 0.4) and Thr (T, RB = 0.4), that display unfavourable properties. Interestingly, at position six, the bulky hydrophobic Ile (I, RB = 0.5) is disfavoured.

Based on the above motif, an in-house repository of peptides was screened for potential binders using a predicted binding score calculated by multiplying the RB values for each position of each peptide. The stability of Acar3 in complex with 94 different peptides indicated as suitable binders, *i.e.* the top scoring peptides which are most likely to represent MHC-I binding peptides, by the PSCPL analysis was measured using SPA. From this assay, the three top-ranked peptides with respect to half-lives of Acar3 complexes, *i.e.* peptide 1 (AMSAQAAAF, “P1”; T½ = 11.3 h), peptide 2 (YMTLQAVTF, “P2”; T½ = 11.2 h) and peptide 3 (MTMITPPTF; “P3”; T½ = 10.3 h) (**Table S1**), were selected for further studies.

### Peptide binding to Acar3 is governed by three footprints

To investigate the structural properties of Acar3, the Acar3-heavy chain (hc) and β_2_m were expressed in *Escherichia coli*, whereafter the Acar3-hc was refolded from inclusion bodies in the presence of soluble β_2_m and one of the three peptides (P1-P3), followed by crystallisation. High-quality crystal structures were obtained for P2 and P3 in space group P2_1_2_1_2_1_ at resolutions of 2.15 Å and 2.25 Å, respectively (**Table 1**, **Figure S3**). The solutions contained one peptide-Acar3 complex in the asymmetric unit, which consists of the Acar3-hc, β_2_m and either of the two peptides.

**Table 1:** Data collection and refinement statistics of Acar3·β_2_m in complex with peptide 2 and 3 (P2, P3).

	[Acar3·β_2_m·P2]	[Acar3·β_2_m·P3]
Data collection		
**Collection source**	EMBL P14	SLS X06SA
**Space group**	P2_1_2_1_2_1_	P2_1_2_1_2_1_
Unit cell parameters		
**a/b/c (Å)**	39.20/48.78/202.13	50.79/65.98/122.84
**α/β/γ (°)**	90.00/90.00/90.00	90.00/90.00/90.00
**Resolution (Å) ^a^**	67.38 - 2.15 (2.21 - 2.15)	46.94 - 2.25 (2.32 - 2.25)
**Total reflections**	286,525	119,270
**Unique reflections**	22,044	20,176
**Completeness (%) ^a^**	99.9 (99.8)	99.5 (99.7)
**Redundancy ^a^**	13.0 (13.4)	5.9 (5.7)
**R_merge_ (%) ^a^**	12.8 (136.7)	13.0 (111.4)
**R_pim_ (%) ^a^**	5.2 (55.5)	8.3 (74.9)
**<I/σ(I)> ^a^**	12.1 (1.9)	8.1 (1.7)
**CC(1/2) ^a^**	0.999 (0.848)	0.995 (0.418)
Refinement		
**R_cryst_ (%)**	17.98	19.75
**R_free_ (%)**	22.94	23.10
**Total number** **of atoms**	3338	3177
Average B-values (Å^2^) and (# of atoms)		
**Acar3_hc**	53.6 (2,212)	58.7 (2,184)
**β_2_m**	51.9 (872)	59.1 (841)
**Peptide**	43.6 (75)	62.8 (71)
**Ligand/ion**	60.3 (10)	81.7 (5)
**Water**	50.7 (169)	53.1 (76)
**r.m.s.d. from ideal**		
**Bond length (Å)**	0.0128	0.0134
**Bond angles (°)**	1.8366	1.9050
Ramachandran plot (MolProbity)		
**Favored (%)**	96.88	96.85
**Allowed (%)**	2.86	2.89
**Outliers (%)**	0.26	0.26
**PDB code**	**7ZQI**	**7ZQJ**

a Values in parentheses are for the highest resolution shell.

**Figure 2:**
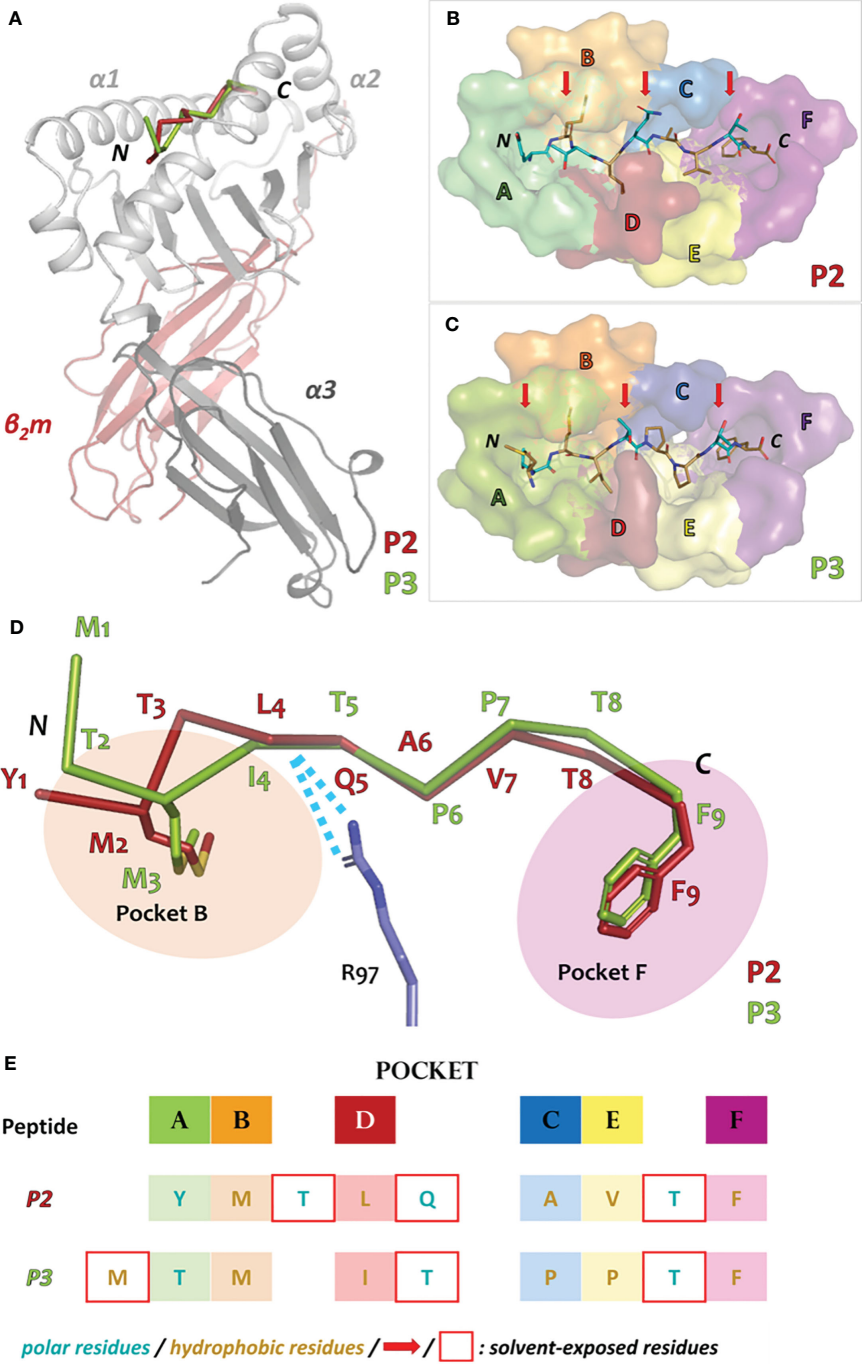
Overall architecture of the Acar3 MHC-I complex and pockets of the Acar3 peptide-binding groove (PBG) and peptide-binding mode. **(A)** Quaternary structure of the Acar3 complex with α1 and α2 represented in light grey, α3 in dark grey and β_2_m in red. The ribbon traces of the two peptides are shown in the PBG created by α1 and α2 (P2: red, P3: green). **(B, C)** Conformations of the peptides (shown as sticks) when bound to Acar3. Hydrophobic residues are shown in sand and polar residues are coloured teal. Solvent-exposed residues are indicated by red arrows. The pockets are shown as surfaces and coloured according to **Figure S4**. **(D)** Schematic representation of the three major anchor points within the Acar3 PBG. The peptides are shown as ribbons (P2: red, P3: green) and the side chains of the two anchor residues are represented as sticks. Arg97 from Acar3 is shown in stick representation in blue and interactions with the peptide backbones are indicated as blue dashes. **(E)** Schematic overview of buried (boxes coloured according to pockets) and solvent-exposed (indicated by a red frame) residues of the two peptides. The tabular arrangement illustrates the aa shifts of the peptides’ residues relative to each other upon binding to Acar3.

The overall arrangement of the Acar3 complex resembles the commonly observed assembly of MHC-I complexes (**Figure 2A**). Six distinct pockets (A-F), previously identified in human HLAs (36), are present in the Acar3 peptide-binding groove (PBG), which is created by the α1 and α2 subunits with their α-helices lining the sides, and the shared β-sheet that forms the bottom of the crevice (**Figures 2B, C**; **S4**).

**Figure 3:**
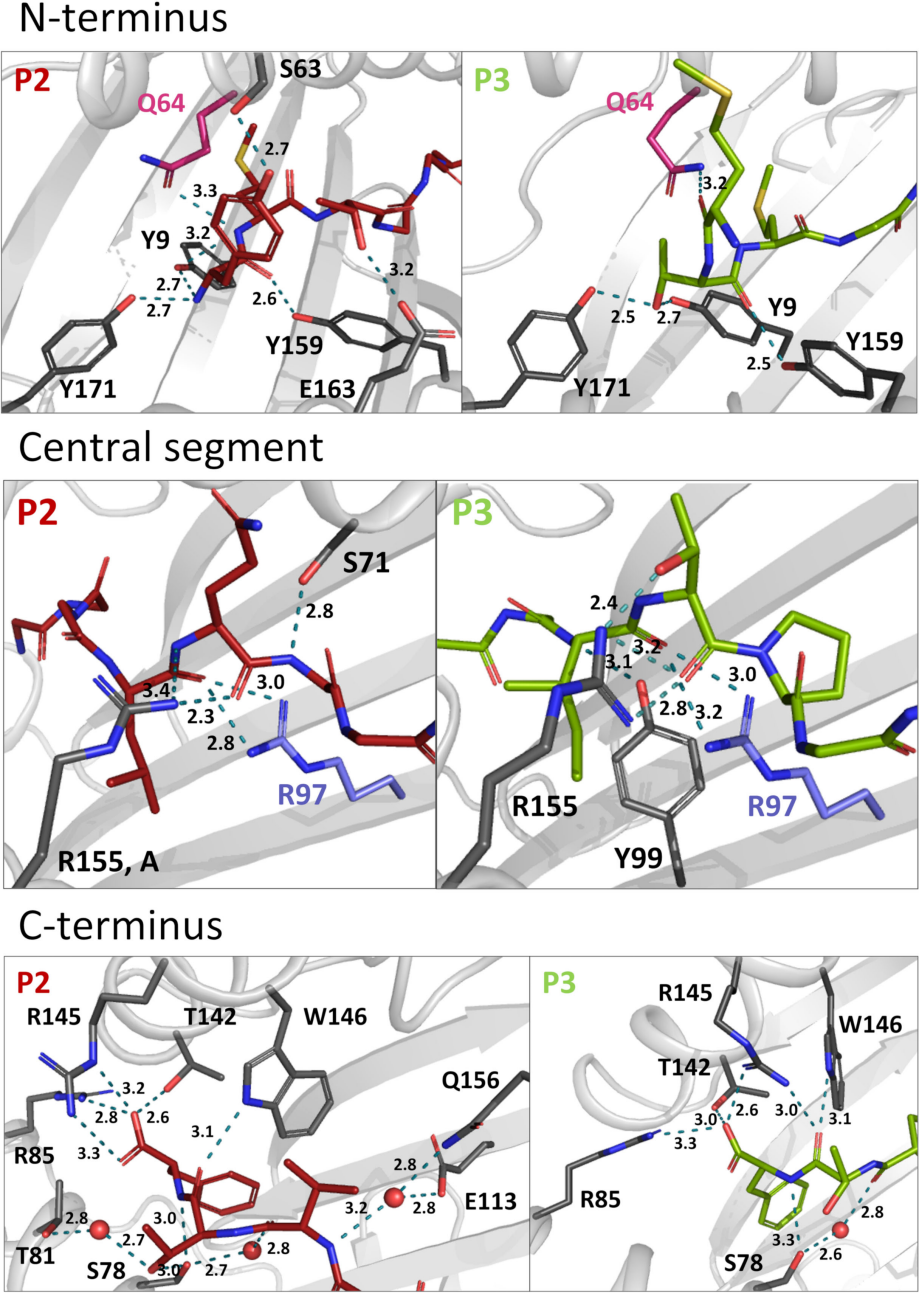
Hydrogen bonds of P2 and 3 within the Acar3 PBG. N-terminal (top row), central (middle row) and C-terminal (bottom row) interactions are shown for P2 (red sticks) and 3 (green sticks). Residues that are involved in hydrogen bonds are shown in dark grey stick representation, and hydrogen bonds are indicated as teal dashed lines. Gln64 and Arg97 are highlighted in pink and blue, respectively. Water molecules are shown as red spheres and distances are given in Å. The side chains of peptide residues more distant from the relevant area have been omitted for clarity. A full list of interactions between P2 and P3 with Acar3 can be found in **Tables S2, S3**.

Comparing the binding modes of peptides P2 and P3 within the Acar3 PBG, it is evident that the five residues at positions 5-9 are well aligned in both structures, whereas their conformations differ substantially among their first four amino acids (**Figures 2D, E**). The Met anchor at position 2 of P2 is placed in pocket B of the Acar3 PBG. Simultaneously, the N-terminal Tyr_1_ residue is accommodated in the aromatic/hydrophobic environment created by the residues of pocket A (**Figure 2**). The N-terminus and the carbonyl oxygen of residue 1 establish hydrogen bonds with the three conserved Tyr residues (Tyr9, Tyr159, Tyr171) in pocket A (**Table S2**; **Figure 3**, top row). Conversely, the Met residue at position 3 (Met_3_) of P3 is positioned in pocket B which causes Thr at position 2 (Thr_2_) of the peptide to be accommodated in pocket A instead of the N-terminus. Consequently, the Thr_2_-side chain mimics the usual N-terminal interactions of antigenic peptides through engaging in a tight hydrogen-bond network with Tyr9 and Tyr171 (**Table S3**; **Figure 3**, top row). The N-terminus and side chain of Met_1_ stick out of the PBG to be solvent-exposed (**Figures 2C–E**). The side chain of Gln64 was found to play a prominent role in facilitating the unusual arrangement of P3 in the Acar3 PBG. While in the P2 structure Gln64 forms a hydrogen bond with the peptide-nitrogen of Met_2_, Gln64 creates a hydrogen bond with the carbonyl oxygen of M_1_ in the P3 complex (**Figure 3**, top row; **Table** **S2, S3**). The different orientation of the Gln64-side chain additionally decreases the negativity of the surface potential of pocket A, and thus contributes to the stabilisation of alternative N-terminal peptide conformations. In contrast to the elongated conformation of P3 resulting from the shifted position of the N-terminal Met-anchor residue, P2 adopts a bent conformation between the third and fifth residue (**Figure 2D**) with Thr_3_ being oriented towards the solvent, while Leu_4_ is inserted into pocked D (**Figures 2B, E**).

Following the conformational differences in the N-terminal segment, both peptides align starting from their residues at position 5. This convergence is facilitated through hydrogen-bonding interactions of the backbone-carbonyl oxygens of Leu_4_ in P2 and Ile_4_ in P3 with the guanidinium group of Arg97 which ascends from the bottom of the PBG (**Figure 2D**; **Figure 3**, middle row). Arg97 acts as a central, unspecific anchor of the peptide backbone and adopts an upright conformation, pointing directly towards the peptide. The orientation of Arg97 towards the centre of the PBG is greatly determined by the surrounding residues: In Acar3, the positioning of Arg97 is stabilised by interactions with its neighbouring residues Asp11 and Glu113 (**Figure S5A**). The combination of the three residues Asp11-Arg97-Glu113 forming a hydrogen-bond network does not occur in any HLA-I molecule. In HLA-B*15:01, which is closely related to Acar3 (discussed later) Arg97 is oriented towards the opposite direction and interacts primarily with Tyr74 (Tyr75 in Acar3), Asp114 (Glu113 in Acar3) and the C-terminal residue of the peptides that are presented by HLA-B*15:01 (**Figure S5B**). The proximity of Arg97 to pocket C in Acar3 may impact the preference for certain residues in the central part of the peptide as seen in the peptide-binding logo (**Figure 1**), such as the hydrophobic residues at position 6 of P2 (Ala_6_) and P3 (Pro_6_).

Residues Val_7_ (P2) and Pro_7_ (P3) are located in pocket E (**Figure 2E**), and the backbone-nitrogen atom Val_7_ participates in water-bridged hydrogen bonds with Glu113/Gln156 (**Figure 3**, bottom row). In both Acar3-peptide complexes, the aromatic side chain of Phe_9_ is deeply buried in pocket F with the backbone-NH and the carboxy terminus oriented towards the opening of the PBG. The Phe_9_-side chain participates in hydrophobic contacts with the pocket-F residues as well as in π-stacking interactions with Phe115 and Phe122. The C-terminal carboxy group of P2 establishes strong hydrogen bonds with Arg85 and Thr142, and slightly weaker hydrogen bonds with Arg145. Moreover, the backbone-NH of Phe_9_ (P2) forms a hydrogen bond with Ser78. P3 forms similar hydrogen bonds to Acar3 as observed for P2, except for the interactions with Arg145 and Thr81 (**Figure 3**, bottom row; **Table S2, S3**).

Overall, the peptide binding signature in Acar3 is determined by three structural features: 1) the adaptability of pocket A and the spaciousness of pocket B (discussed below) to permit great flexibility of the N-terminal part of the peptides, 2) the central backbone anchor Arg97 that imposes conformational uniformity on the C-terminal backbone trace of bound antigens, and 3) the narrow and specific pocket F, which allows tight binding of the C-terminal anchor residue of the peptides (**Figure 2D**).

### A unique composition of pocket B residues permits great N-terminal flexibility of peptides bound to Acar3

**Figure 4:**
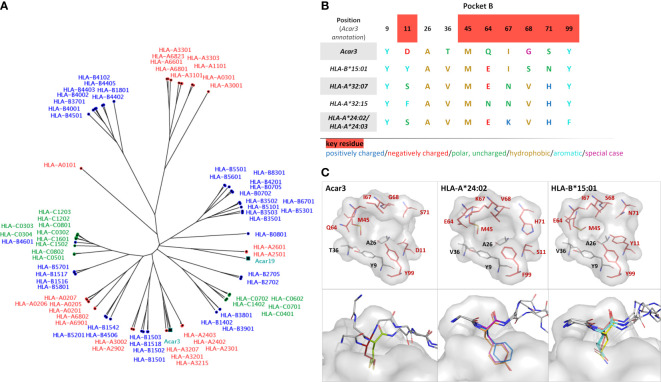
Comparison of Acar3 with phylogenetically close HLA-I molecules. **(A)** Phylogenetic tree including Acar3, Acar19 and HLA class-I molecules. **(B)** Comparison of pocket B residues of Acar3 with its closest HLA-I molecules. Key-residue positions are indicated in red. (**C**, top row) The pockets from **(B)** shown as surface (grey) and stick representation with key residues coloured red. (**C**, bottom row) Positional variance of anchor residues accommodated in pocket B of Acar3, HLA-A*24:02 and HLA-B*15:01. The pocket is shown as grey surface, the peptides are shown as sticks with the anchor residue in colour and the surrounding residues shown in grey and without side chains. For Acar3, the anchor residues are depicted in red (M_2_ (P2)) and green (M_3_ (P3)). From the structures of HLA-A*24:02 and HLA-B*15:01 available in the PDB, three representatives have been chosen each. HLA-A*24:02: 2BCK, chain A, B and C (Y_2_ in orange), 3I6L (F_2_ in blue) and 4F7M chain A, B and C (Y_2_ in magenta); HLA-B*15:01: 1XR8 (E_2_ in salmon), 1XR9 (L_2_ in cyan) and 5VZ5 (Q_2_ in yellow).

A search for P1, P2 and P3 sequences in the Immune Epitope Database (IEDB) revealed that all three peptides were found to bind different HLA-I molecules in previous studies. Applying either a minimum peptide-MHC class I affinity of 500 nM (37, 38) or a minimum peptide-MHC class I half-life of 1 h (39) as threshold, we found that P1 binds HLA-B*15:01 (40, 41), while P2 shows affinity to a broader range of HLA-I molecules, such as HLA-A*24:03, HLA-B*15:01 and HLA-B*35:01 (40–42). Studies of P3-binding to different HLA-I molecules revealed an even broader spectrum of binding partners, *i.e.* HLA-A*32:07, HLA-A*32:15, HLA-A*68:23, HLA-B*35:01 and HLA-B*58:01 (40, 41, 43). To evaluate which of the HLA molecules that bind P1, P2 and P3 obtained from the IEDB are most similar to Acar3, we prepared a distance tree based on peptide-binding motifs using *in-silico* MHC-binding predictions (*MHCcluster, see Materials and methods*) (44) (**Figure 4A**). In short, binding to the included MHC-I molecules (Acar3 and Acar19) was predicted for a set of random natural peptides using a version of *NetMHCpan* retrained to include a small set of peptides with measured half-lives for the two Acar molecules. Next, the functional similarity between any two MHC-I molecules was defined from the correlation of the union of the predicted top 10% of strongest binding peptides for the two MHC-I molecules, and the distance matrix used to calculate the functional tree. The four most closely related HLA-I molecules to Acar3 with respect to the distance tree, which bind at least one of the three peptides, are HLA-A*32:07, HLA-A*32:15, HLA-A*24:03 and HLA-B*15:01. Peptide-binding logos from more distant HLA molecules (available on the *NetMHCpan 4.0 Motif Viewer* website) which were found to bind P2 and/or P3, showed either ambiguous or inconclusive residue preferences at position 2 or a preference for Thr, which occupies the second position of P3 and the third position of P2. An alignment of the residues (including key residues) that flank pocket B (45) in Acar3 and the five HLA-I molecules reveals a unique amino-acid signature of this pocket for Acar3 (**Figure 4B**). The key residues define the shape and depth of the pockets and establish interactions with the peptides’ anchor residues. HLA-I molecules most closely related to Acar3 whose structures are available in the PDB comprise HLA-A*24:02 and HLA-B*15:01. HLA-A*24:02, despite showing weak binding to P2 and P3, is used as a structural substitute for HLA-A*24:03, since the (key) residues of pockets B and F in both molecules are identical, and the only difference in the PBG between the two molecules is the substitution of two amino acids (Asp166/Gly167 in HLA-A*24:02 *vs.* Glu166/Trp167) in pocket A. Both HLA-A*24:02 and HLA-B*15:01 reveal a rather narrow and elongated shape of pocket F similar to Acar3, thereby permitting little conformational flexibility of the C-terminus. In contrast, compared to HLA-A*24:02 and HLA-B*15:01, pocket B of Acar3 is wider and more open towards the PBG, which is the result of small residues at positions 11 (Asp) and 71 (Ser), segregating pocket B from pocket C (**Figure 4C**, top row). Moreover, with respect to all known classical and non-classical HLA class-I molecules, a Gly residue at position 68 (67 in HLA) is a unique feature of Acar3 and has particular impact on the depth and width of pocket B. In Acar3, the peptide’s anchor residue accommodated in pocket B has thus greater conformational freedom which results in an increased flexibility of the N-terminal half of the peptide. In addition to Met as the preferred anchor residue, pocket B provides sufficient space to accommodate residues of secondary importance, *e.g.* Trp, as occurring in the peptide-binding logo (**Figure 1**). In contrast, the anchor residues located in the B pockets of HLA-A*24:02 and HLA-B*15:01 show less positional deviation among different structures – even when anchor residues of different chemical properties and peptides of different lengths are considered (**Figure 4C**, bottom row). This is reflected by the root-mean-square deviation (r.m.s.d.) values among the N-terminal regions (aa 1-3) of the peptides bound to Acar3 being twice as high as in HLA-A*24:02 and HLA-B*15:01-peptide complexes. In contrast, the relative flexibility among the central amino acids (aa 4-6) of peptides bound to HLA-A*24:02 and HLA-B*15:01 is significantly higher (≈ 2-fold) than among Acar3-bound peptides (**Table S4**).

## Discussion

Great reed warblers are long-distance migratory songbirds that successfully handle pathogens at breeding, stop-over and wintering sites (46). It has been suggested that their highly duplicated MHC-I genes are associated with particularly well-developed immune adaptations (47). Our work presents the first structure of an MHC-I molecule from a wild songbird in complex with two different antigenic peptides and elucidates the structural basis of a unique peptide-binding mode. Although the overall architecture of the Acar3 peptide-binding groove comprising six distinct pockets closely resembles the characteristics of HLA class-I molecules, Acar3 does not completely resemble any supertypes defined for HLA-I molecules (45). Together with the unique amino-acid composition and shape of pocket B, the central Arg97 residue strongly contributes to defining the Acar3-peptide binding mode. Whereas Arg97 draws the central part of the bound peptides closer to the bottom of the PBG, thereby contrasting the conformation usually observed in peptide-HLA-I complexes, pocket B enables an outstanding N-terminal flexibility with Gln64 permitting a shifted arrangement of the peptides’ N-terminal part. This observation puts the necessity of a Glu residue at position 63 in porcine MHC I (SLA-1*0401) and in HLA class I (corresponding to Gln64 in Acar3) for the N-terminal extension presentation mode as proposed by a recent study (48) into perspective, and suggests that N-terminally extended peptide binding can be facilitated in different ways across vertebrate species. With respect to the possibility for either Met_2_ or Met_3_ to be accommodated in pocket B of Acar3, it is likely that positions 2 and 3 in the peptide-binding logo (**Figure 1**) represent a combined anchor rather than two distinct positional amino-acid specificities. In addition to the preference for Met at both positions, pocket B provides sufficient space to accommodate a Trp residue. The slightly negative surface potential of the cavity could furthermore permit the insertion of Lys and Arg, which represent subordinate anchor residues at position 2 of the peptide-binding logo. The increased residue preference at position 6 of the peptide-binding logo possibly results from the role of Arg97, which anchors the bound peptides to the centre of the PBG. However, the different properties of the amino acids at position 6, *i.e.* Pro, Glu and Ala, probably reflect the unspecific nature of this interaction through the formation of hydrogen bonds with the peptide backbone.

The preferred anchor residues assigned to pocket B, Met and – to a lesser extent – Trp, usually occur at low frequency in the proteomes of living organisms, including potential pathogens (49–54) (**Figure S6**). Those residues are among the amino acids with the highest metabolic synthesis costs (49, 50, 55), which potentially causes them to be present almost exclusively at specific sites within proteins. Many different criteria have been established to predict the mutational probabilities of amino-acid residues in proteins, including protein stability, genetic code and physicochemical similarities of amino acids (56–58). Due to their distinct properties with respect to *e.g.* resilience to oxidative stress (Met) and protein folding (Trp) (49, 59–62), a substitution of those residues within the context of a protein will likely result in less stable – probably even less functional – variants, and thus impose a fitness cost on the pathogen. Therefore, the mutation rate of Met and Trp is comparably low (56, 63), impeding substitutions of potential anchor residues that would prohibit binding of pathogen-derived peptides to Acar3. The conformational variability in the N-terminal halves of Acar3-binding peptides results in two potential positions (2 or 3) of the anchor residue within the peptide sequence. This increases the repertoire of antigens harbouring rare amino acids like Met or Trp that can be presented by Acar3. However, the accommodation of an anchor residue such as Met at position 3 of the peptide in pocket B will probably depend on the presence of a small, polar residue, *e.g.* Thr or Ser, at position 2 acting as a mimic for the N-terminal amino group.

Two recent studies reported on an N-terminally shifted binding mode of the HIV Gag epitope TW10 (TSTLQEQIGW) to HLA-B*57:01 and HLA-B*58:01 (64, 65), closely resembling the non-canonical binding of P3 to Acar3 presented in this work. In both studies, the N-terminal residue of the TW10 peptide is solvent exposed while Ser at position two occupies the actual position of the amino group in pocket A. On the contrary, an HIV-escape mutant, TW10-T3N (TS**N**LQEQIGW), leads to a canonical binding mode of this peptide variant with Thr at position one being accommodated in pocket A of HLA-B*57:01. The N-terminal register shift causes major conformational changes in the C-terminal part of the bound peptide, thereby attenuating antigen recognition of the escape mutant by the NK receptor KIR3DL1 (64). Intriguingly, in Acar3 no such C-terminal conformational changes caused by the N-terminal register shift can be observed, which emphasises the particular role of the central Arg97 acting as an unspecific anchor for the peptide backbone. The non-distinctive nature of this central anchoring point with respect to peptide sequence renders the antigen presenting system less susceptible towards escape mutations in pathogens. Hence, the unique peptide-binding mode of Acar3 could have important implications for maintaining the antigen presentation and for hindering immune evasion by pathogens: Both, the expansion of the peptide repertoire through permitting N-terminal flexibility, as well as putative pathogen escape mechanisms may not impair antigen recognition by NK receptors that interact with the C-terminal part of the presented peptide. These include receptors with similar functions to killer immunoglobulin-like receptors (KIR) which have been identified in many vertebrate species (66–69), and are thus likely to be present also in songbirds.

Although the majority of peptides presented by MHC molecules are of self-origin, this is unlikely in the case of the three top-binding peptides to Acar3, since they were not found within the great reed warbler exome. It is therefore more likely that they stem from either viral or bacterial pathogens capable of invading cells, which explains their presentation on MHC class-I molecules rather than MHC-II molecules. Using the example of the role of HLA-B*57 in HIV, it has been demonstrated that the interplay between the innate immune response mediated by KIR3DL1 recognition and the adaptive immunity through the activation of CD8^+^ T cells enhances the protection against an unfavourable disease outcome (64, 70). Acar3 may thus have a role at the interface of innate and adaptive immunity and could be an example of a sophisticated mechanism in migratory birds to counter immune evasion of different pathogens.

The current study allows an initial glimpse of how songbirds with numerous MHC-I genes present antigens for pathogen recognition. Little is known about the abundance and composition of particularly pathogen-derived antigenic peptides in the great reed warbler. Hence, the investigation of peptide binding to Acar3 in this study is partly limited by the use of a peptide library derived from human pathogens. As a consequence, certain combinations of amino acids within antigenic peptides that may be specific to the great reed warbler could be missing or underrepresented, while on the other hand, other sequences that are more common in human pathogen-derived peptides may be overrepresented. In line with this, the functional clustering of Acar3 within the distance tree may be biased by the wealth of data available on antigen binding to HLA I compared to a lesser amount of data derived from other organisms. A next step could be to identify pathogen-derived antigens that originate from the great reed warbler and test the stability of the corresponding peptide-MHC-I complexes. With that knowledge at hand, more structural data could be generated – also including several different MHC-I variants – to get to the bottom of the putative benefit behind the large number of MHC-I genes in the great reed warbler and other songbirds. Such analyses would verify if the N-terminal flexibility of antigens that we observe in this study represents a common theme not only in Acar3 but also in other MHC-I variants; also with respect to the limited insight that is provided by the structural data from only two peptides.

## Materials and methods

### Amino acid sequences of Acar3, Acar5, Acar19 and Beta-2-microglobulin

The Acar3, Acar5 and Acar19 heavy-chain (hc) sequences have been published previously (31, 71). To amplify the *β* ***_2_*** *m* gene, degenerated PCR-primers were designed from transcriptomic *β* ***_2_*** *m* sequences in willow warbler (*Phylloscopus trochilus*, NCBI SRA Accession no. SRA056327 (72), house sparrow (*Pado*, NCBI SRA Accession no. SRP012188 (73),) and zebra finch (*T. guttata*, GenBank accession no; DQ215661 and DQ215662 (74),). A 533-bp long *β_2_m* fragment spanning the whole mature peptide region and partially the 3’ UTR region was amplified with the degenerated forward primer P162 5’ CGGGGWGCCCTGGCGCTC 3’ and the reverse primer P149 5’ GGCCTGCAGACCTCCCTTGA 3’ using *Acar* cDNA template from two great reed warbler individuals. Each PCR reaction contained 2 µl diluted cDNA (for details on RNA extraction and cDNA preparation (75)), 0.2 µM of each primer, 1.5 mM MgCl_2_, 1.25 U AmpliTaq DNA Polymerase and 1x GeneAmp Buffer II (Applied Biosystems) in a total volume of 25 µl. The following cycling parameters were used: 94°C for 3 min and then 35 cycles of 94°C for 30 s, 58°C for 30 s and 72°C for 90 s. A final extension step at 72°C for 15 min was applied. PCR products were Sanger sequenced in the forward and reverse direction using standard procedures (BigDye terminator Cycle Sequencing kit v.3.1, Life Technologies) on an ABI PRISM 3130 genetic analyser (Applied Biosystems). The resulting sequences were inspected and manually edited with the software Geneious (v.5.5, Biomatters). A 495 bp-long partial *Acar β* ***_2_*** *m* transcript was successfully sequenced from great reed warbler cDNA (*Acar β_2_m*, GenBank Accession no. KM096440). No polymorphic sites were detected within or between the two sequenced individuals. The *Acar-β* ***_2_*** *m* fragment included partial signal peptide information (39 bp), the whole predicted mature peptide (297 bp) and partial 3’ UTR (156 bp).

### Protein production (heavy chain (hc) and β_2_m) for Scintillation Proximity Assays (SPA)

*Acar* MHC-I heavy chains (residues 1-272) containing a C-terminal histidine-affinity tag for purification and biotinylation substrate peptide (BSP) for biotinylation were produced recombinantly and purified as previously described (76). In brief, the proteins were overexpressed in *Escherichia coli*, resulting in the formation of inclusion bodies. After mechanical cell lysis, inclusion bodies were isolated by centrifugation and dissolved in 8 M urea, 25 mM Tris/HCl, pH 8.0, and purified by immobilised metal (Ni^2+^) affinity chromatography (IMAC), hydrophobic interaction chromatography and size exclusion chromatography. *In-vivo* biotinylation of the BSP-tagged MHC-I hc was achieved by co-expression of the BirA enzyme.

*Acar* β_2_m containing an N-terminal histidine-affinity tag and a factor Xa cleavage site, was produced by overexpression in *Escherichia coli* and the inclusion bodies were isolated and dissolved in 8 M urea as described above. The protein was first purified by IMAC in 8 M urea, 25 mM Tris/HCl, pH 8.0. The purified and denatured β_2_m was folded by drop-wise dilution into a non-denaturing buffer (300 mM urea, 25 mM Tris/HCl, pH 8.0) over 24 hours. The folded β_2_m protein was then treated with Factor Xa to remove the N-terminal affinity tag. Thereafter, cleaved β_2_m was separated from the uncleaved fraction and the affinity tag by IMAC, and lastly purified by size-exclusion chromatography (SDX200-PG).

### Generation of a 9-mer positional scanning combinatorial peptide library (PSCPL)

Positional scanning combinatorial peptide libraries (PSCPL) were synthesised as previously described (77). Peptide-binding specificity of MHC-I resolved into an array of apparently independent sub-specificities: quantitation by peptide libraries and improved prediction of binding. Briefly, eight of nine positions comprised an equimolar pool of 19 of the 20 natural amino acids (*i.e.* excluding cysteine), whereas the remaining position comprised 1 of the 20 natural amino acids (*i.e*. including cysteine), thereby interrogating the position-specific effect of this latter amino acid. In one synthesis, the amino-acid pool was used in all nine positions. The library therefore consisted of (20 x 9) +1, or 181 individual peptide libraries, where X denotes the random incorporation of an amino acid from the mixture, and the fixed amino acid and its identity is indicated by the single letter amino-acid abbreviation: 20 PSCPL sub-libraries describing position 1, AX8, CX8, DX8,….YX8; 20 PSCPL sub-libraries describing position 2, XAX7, XCX7, XDX7,…. XYX7; and so forth to 20 PSCPL sub-libraries describing position 9, X8A, X8C, X8D,…. X8Y; and finally, a completely random peptide library, X9 (*for further details see* (34, 77, 78)). The PSCPL approach exploits the fact that MHC class I tends to bind peptides “in register”, which means that each position of any of the many random peptides of a 9-mer PSCPL is “offered” to the same MHC pocket. Thus, we assume that the binding of a sub-library with a “locked” amino acid in a certain position reflects the specificity of the complementary MHC-I pocket, and that the complete PSCPL represents the specificity of an MHC-I molecule in question showing preferred and disfavoured amino-acid residues (as well as amino acid residues of no importance, specificity-wise) corresponding to each position of the 9-mer peptide ligands. *A priori*, a good binder would be one that represents preferred amino acids corresponding to the primary and secondary anchor positions of the MHC-I in question.

### Scintillation Proximity Assay (SPA) based peptide-MHC-I dissociation assays

Both to determine the peptide length preference of two different MHC-I molecules, Acar19 and Acar5, and the binding motif of Acar19 and Acar3, peptide-Acar (pMHC-I) dissociation was measured using a scintillation proximity assay (SPA) as described in (34, 78).

The SPA assay is a proximity-based assay, which allows real-time, on-line detection of binding. The signal is generated when the iodinated β_2_m is in proximity of the streptavidin- and scintillant-coated surface of the assay plate. This proximity is dependent upon the peptide-dependent binding of radiolabelled β_2_m to the biotinylated MHC-I heavy chain, the latter being bound to the streptavidin. Whenever a peptide dissociates off the MHC-I, the β_2_m also dissociates off and the signal abates. This assay is uniquely suited to describe peptide-MHC-I stability.

To determine the preferred length, we evaluated peptide binding in terms of the amounts of complexes formed with peptides of different lengths in an unbiased way, and thus employed peptide libraries ranging from 7 to 13 residues in length. The motif characterisation in this study only focuses on Acar3, and the Acar3-complex stability was determined for nonameric peptides from the 9-mer PSCPL using SPA (76, 78). Briefly, denatured and biotinylated Acar3-hc was diluted into PBS/0.1% Lutrol^®^ F68 containing 10µM of peptide and trace amounts of ^125^I radiolabelled β_2_m in streptavidin coated scintillation microplates (FlashPlate^®^, Perkin Elmer, SMP103001PK or SMP410001PK). Plates were incubated over night at 18°C to attain complex folding. Dissociation was initiated at time point zero (Y_0_) by addition of unlabelled β_2_m and scintillation was measured continuously for up to 24 hours at 37°C in a TopCount NXT liquid scintillation counter (Packard). The resulting dissociation data for each peptide sub-library was used to calculate a relative binding value (RB-value) by dividing the approximated area under the curve (AUC) of the sub-library with the AUC of the reference library according to the following equation: RB = AUC_sub-library_/AUC_X9_. The RB values of each amino acid in a given position were summarised and normalised so the sum equals 20 for each position. A matrix using the RB-values was then generated where an RB-value ≥ 2 defines a favoured amino acid at a specific position, and an RB-value of ≤ 0.5 defines a disfavoured amino acid at a specific position (77, 78). Thus, the matrix defines the peptide-binding properties of the Acar3 molecule. The logo of the PSCPL-derived Acar3-binding motif was made using the Seq2Logo 1.1 server as P-Weighted Kullback-Leibler logos (35).

Using the matrix obtained from the PSCPL, a predicted Acar3-binding score can be calculated for a given 9-mer peptide sequence by multiplying the RB values for each position of a specific peptide, *e.g.* (aa Pos. 1 RB)*(aa Pos. 2 RB)*(aa Pos. 3 RB)*(aa Pos. 4 RB)*(aa Pos. 5 RB)*(aa Pos. 6 RB)*(aa Pos. 7 RB)*(aa Pos. 8 RB)*(aa Pos. 9 RB) (77). The rank score was calculated for some 9500 in-house 9-mer peptides and the top 94 scoring peptides were selected for binding studies using SPA as described above. The three peptides that yielded pMHC-I complexes with the highest stability in the SPA were peptide 1 (P1, AMSAQAAAF), peptide 2 (P2, YMTLQAVTF) and peptide 3 (P3, MTMITPPTF) (**Table S1**).

### Functional clustering

The *Acar* MHC-I and human HLA-I allomorphs were clustered using the *MHCcluster* method (79), which predicts and functionally clusters the peptide-binding specificities of the MHC-I allomorphs. The *MHCcluster* method used here was based on the retrained version of the *NetMHCpan* method (80), trained including a small set of peptides with measured half-lives for Acar3 (188 peptides) and Acar19 (275 peptides) complexes (**Table S5**). *MHCcluster* estimates the functional similarities between any two MHC-I allomorphs in the analysis by correlating the union of the predicted top 10% of strongest binding peptides for each of the defined allomorphs. If two MHC-I allomorphs were predicted to have a perfect overlap regarding their peptide-binding specificities, the similarity was defined as 1, and if there was no overlap at all, the similarity was defined as 0. The distance matrix was converted to a distance tree using UPGMA clustering. To estimate the significance of the MHC-I distance tree, 1000 distance trees were generated using the bootstrap method. The bootstrapping was performed at the peptide level, *i.e.* for each distance tree a new set of 100.000 peptides and the correlating prediction was selected from the original pool of 100.000 peptides with replacements. The trees were then summarised, and a consensus tree was made with branch bootstrap values.

### Protein production and refolding for structural studies

The Acar3-hc (residues 1-272) was cloned into a pET26b(+) vector, and electrocompetent *E. coli* TUNER (DE3) cells were transformed with the plasmid. Subsequent protein expression was performed using BD Difco LB broth Miller supplemented with 50 μg/ml kanamycin. Cells were grown at 37°C, 120 rpm, and when an OD_600_ of 0.8 was achieved, IPTG was added to a final concentration of 1 mM. Four hours after induction, cells were harvested, and the pellets were stored at -80°C. The inclusion bodies containing the Acar3-hc, were isolated from the cells and washed according to previously published protocols (81, 82). Finally, the inclusion bodies were solubilised in 20 mM Tris/HCl, pH 8.0, 8 M urea, 1 mM EDTA, 10 mM DTT (hereafter referred to as “IB buffer”) and stored at -80°C until further use.

A synthetic *β_2_m* gene that was codon-optimised for *E. coli* was ordered from GenScript and cloned into the pET-26b(+) vector. The gene was then moved to the expression vector pNIC28-Bsa4 using ligase independent cloning (LIC). The resulting pNIC28-b2m construct encodes β_2_m with an N-terminal (His)_6_-tag that can be removed by TEV cleavage, leaving a single N-terminal Ser on β_2_m. Electrocompetent *E. coli* TUNER (DE3) cells were transformed with the plasmid and expression was carried out in TB medium supplemented with 50 μg/ml kanamycin. The culture was started at 30°C, 120 rpm, and at an OD_600_ of 0.5, the temperature was lowered to 18°C. At OD_600 =_ 0.9, IPTG was added to a final concentration of 0.1 mM. The cells were harvested 20 hours after induction and the pellets were solubilised in 50 mM Tris/HCl, pH 8.0, 300 mM NaCl, 20 mM imidazole. Cells were disrupted mechanically through sonication or application of pressure and the lysate was applied to a Ni^2+-^affinity column using a gradient of 20-500 mM imidazole. Afterwards, the protein was purified by size-exclusion chromatography (SEC) in 20 mM Tris/HCl, pH 8.0, 150 mM NaCl, 1 mM EDTA, concentrated and stored at -80°C until further use.

Purified β_2_m was added to 200 ml of 100 mM Tris/HCl, pH 8.0, 400 mM L-Arg, 1 mM EDTA, 5 mM GSH, 0.5 mM GSSG, 0.5 mM PMSF at 4°C under gentle stirring to a final concentration of 2 μM. Denatured Acar3 (solubilised in IB buffer) was premixed with P2 (solubilised in DMF) or P3 (solubilised in 50% (v/v) water + 50% (v/v) IB buffer) in a concentration that would result in final concentrations of 1 μM Acar3 and 10 μM peptide upon addition to the β_2_m-containing refolding buffer. The peptides used for the refolding experiments were purchased from Peptides International Inc. with a purity level of ≥ 95%. Refolding was initiated by the dropwise addition of 1/3 of the volume of the Acar3-peptide mixture to the β_2_m-containing refolding buffer under continuous stirring. After 12 and 24 h each, again 1/3 of the Acar3-peptide mixture were added and stirring continued. After 48 h of incubation, the refolding mixture was transferred to 4 l of 50 mM Tris/HCl, pH 8.0, 150 mM NaCl, 0.5 mM EDTA and dialysed for 2 h. Then, the mixture was moved to 4 l of 50 mM Tris/HCl, pH 7.5, 150 mM NaCl and dialysed for approx. 10 h. The dialysate was concentrated and applied to a HiLoad 16/600 Superdex 200 column using 20 mM Tris/HCl, pH 7.5, 150 mM NaCl (SEC buffer) and the fractions containing the complex were pooled and concentrated.

### Crystallisation and structure determination of [Acar3·β_2_m] in complex with P2 and P3

Prior to crystallisation, the refolded complexes were diluted in SEC buffer to final concentrations of 5 mg·ml^-1^ ([Acar3·β_2_m·P2]) or 10 mg·ml^-1^ ([Acar3·β_2_m·P3]) and centrifuged for 10 min at 16100 x g. Crystallisation was performed at 293 K, using the hanging-drop vapour-diffusion method at a protein-to-reservoir ratio of 1:1 in the drop. To obtain crystals of sufficient size and quality, the [Acar3·β_2_m·P2] crystallisation drops were subjected to streak seeding, using a seeding solution from previously obtained crystals that were smashed by sonication. Crystals of [Acar3·β_2_m·P2] grew in 100 mM Hepes/MOPS, pH 7.0, 60 mM divalent mix (20 mM MgCl_2_/40 mM CaCl_2_) and 32.5% precipitant mix (25% (v/v) MPD/25% (w/v) PEG 1000/25% (w/v) PEG 3350) and were flash-frozen in N_2_ (l) without cryo-protection. Crystals of [Acar3·β_2_m·P3] were obtained in 100 mM MES, pH 6.0, 6% (v/v) Tacsimate, pH 6.0 and 25% (w/v) PEG 4000, cryo-protected with 35% (v/v) ethylene glycol and flash-frozen in N_2_ (l).

Diffraction data were collected at DESY (PETRA III), EMBL Hamburg, Germany, at beamline P14 ([Acar3·β_2_m·P2]), and at SLS, PSI Villigen, Switzerland, at beamline X06SA ([Acar3·β_2_m·P3]). The data were processed with XDS (83) and further steps were carried out using programs from the CCP4 program suite (84). Molecular replacement was performed with PHASER (85) using an MHC-I molecule from duck (*Anas platyrhynchos*, PDB: 5GJX) as search model for the [Acar3·β_2_m·P3] structure. Thereafter, for [Acar3·β_2_m·P2], the previously solved structure of [Acar3·β_2_m] was used as search model. Structure refinement was carried using REFMAC (86) and phenix.refine (87), and model building was performed with Coot (88, 89).

The protein structures presented in this article has been submitted to the Protein Data Bank (http://www.rcsb.org/pdb/home/home.do) under accession numbers 7ZQI and 7ZQJ.

## Data availability statement

The datasets presented in this study can be found in online repositories. The names of the repository/repositories and accession number(s) can be found below: The protein structures presented in this article has been submitted to the Protein Data Bank (http://www.rcsb.org/pdb/home/home.do) under accession numbers 7ZQI and 7ZQJ.

## Author contributions

Conceptualisation: HW, KL-P, and PK conceived the study. Methodology: SB, MN, KL-P, and HW provided methodologies in their respective expertise. Formal analysis: SE, SM, SB, and MN analysed data related to their respective expertise. Investigation: SE and SB performed the experiments and collected the data. Resources: HW and KL-P provided study material, reagents and materials. Writing: SE provided the first draft of the manuscript and all co-authors contributed with reading, revising and commenting on the manuscript until the final version. Visualisation: SE, SB, and MN prepared the figures and tables. Supervision: HW took the main leadership responsibility for the research activity. Project administration: HW coordinated the responsibility for the research activity planning and execution. Funding acquisition: HW was responsible for the financial support for the project leading to this publication. All authors contributed to the article and approved the submitted version.
