# Supplementary material for: The structure of songbird MHC class I reveals antigen binding that is flexible at the N-terminus and static at the C-terminus
Source: Front Immunol. 2023 Jul 7;14:1209059. doi: 10.3389/fimmu.2023.1209059 (PMC10360169; doi:10.3389/fimmu.2023.1209059)
Supplement: Supplementary file 1 [file DataSheet_1.pdf]

Figure S1

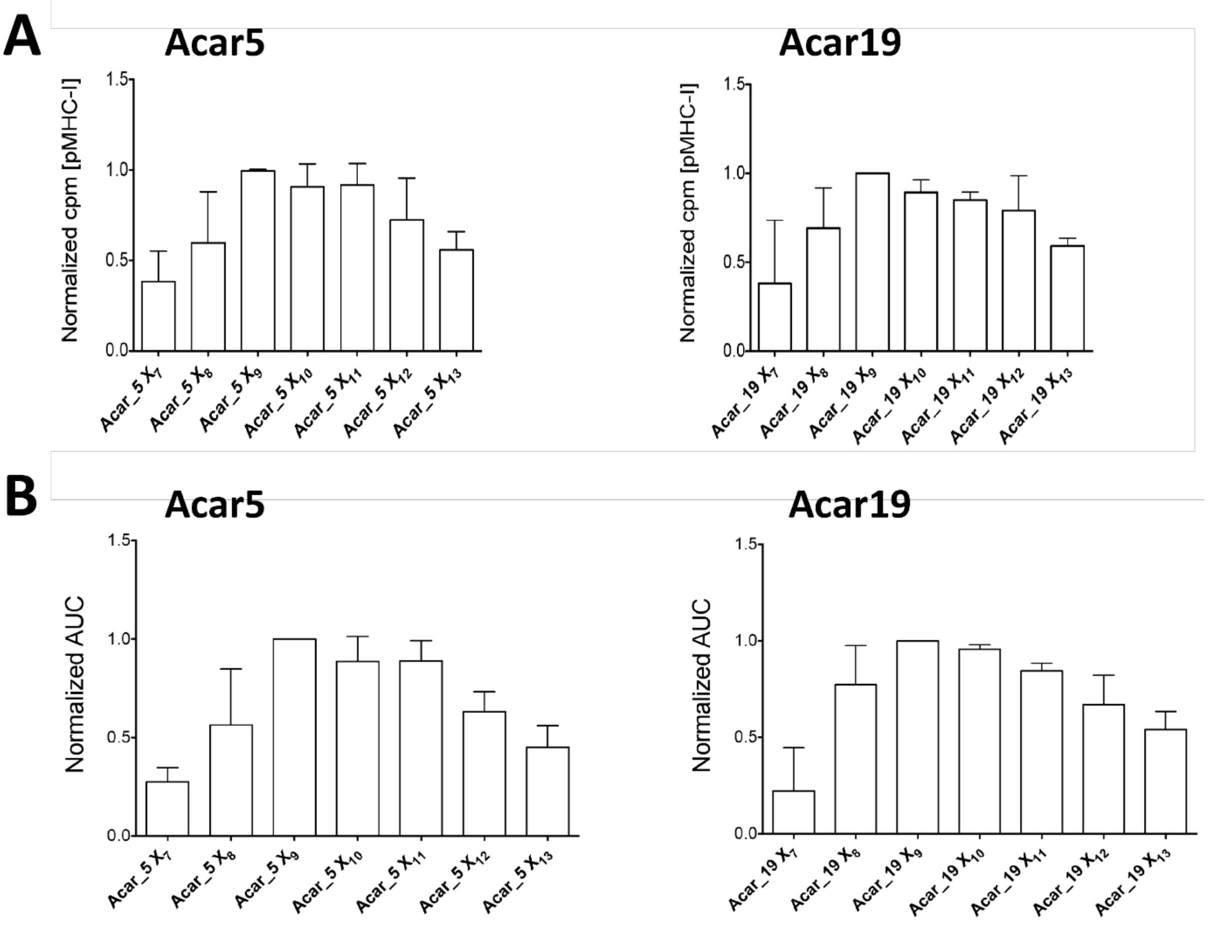

Figure S1: Great reed warbler MHC-I molecules show classical MHC-I peptide-length preferences. The heavy chains (hc) of Acar5 (left) and Acar19 (right) that are closely related to Acar3 (95.3 % and 88.7 % sequence identity, respectively) were folded in the presence of X_7-X_{13} peptide libraries with ^{125}I-labeled *Acar* β_2m. **(A)** The number of complexes formed after 24 hours folding at 18 °C was analysed in a scintillation proximity-based assay (SPA) at 37 °C. Acar5 and Acar19 formed significantly less complexes with 7-mer peptides compared to 9-, 10- and 11-mers. **(B)** The dissociation of Acar pMHC-I complexes formed with X_7-X_{13} peptide libraries was monitored for 24 hours to investigate complex stability. The normalised area under the curve (AUC) was calculated for each Acar-X_n MHC-I allomorph combination and values were compared to Acar-X_9 MHC-I, which formed the most stable complexes with both, Acar5 and Acar19.

Figure S2

Amino acid		Peptide position								
		1	2	3	4	5	6	7	8	9
	**A**	0,9	0,8	0,9	1,0	1,4	1,3	1,1	1,0	0,8
	**C**	0,9	1,0	0,7	0,7	1,2	1,0	1,0	1,0	1,0
	**D**	1,2	1,0	1,1	0,7	0,7	1,1	0,9	1,4	1,3
	**E**	1,1	0,7	0,8	1,1	0,9	1,3	1,3	1,3	1,2
	**F**	0,9	1,1	*0,3*	1,0	1,0	1,0	1,1	1,0	**2,6**
	**G**	1,1	0,6	0,6	1,0	1,0	0,7	0,8	0,7	1,1
	**H**	0,7	0,8	0,8	0,8	0,9	1,1	1,2	1,2	0,8
	**I**	0,9	1,0	1,0	0,9	1,1	*0,5*	0,8	0,8	1,5
	**K**	1,1	1,4	1,2	1,0	1,3	1,0	1,1	0,8	*0,3*
	**L**	1,0	0,7	1,2	1,1	1,1	1,0	1,0	1,1	1,9
	**M**	0,9	1,6	**2,1**	1,3	1,3	1,0	0,9	1,0	1,5
	**N**	1,2	0,7	1,2	1,2	0,8	1,0	1,2	1,0	0,6
	**P**	0,8	1,0	1,0	1,2	1,3	1,8	1,4	1,3	0,6
	**Q**	1,2	1,0	1,0	1,2	1,1	1,2	1,2	0,9	*0,4*
	**R**	0,9	1,3	0,9	1,1	0,7	1,0	1,1	0,7	0,6
	**S**	1,0	0,6	1,0	1,0	0,7	0,6	1,0	0,9	*0,4*
	**T**	0,9	1,2	1,2	1,0	1,1	1,1	0,9	1,0	*0,4*
	**V**	1,1	1,1	1,1	0,8	1,0	1,2	1,0	1,0	1,2
	**W**	1,1	1,2	1,4	1,3	0,7	1,0	1,0	0,8	0,9
	**Y**	1,2	0,9	1,1	0,8	0,8	1,0	1,1	1,0	0,7
	**Sum**	**20**	**20**	**20**	**20**	**20**	**20**	**20**	**20**	**20**
	**AP**	0,6	1,5	2,5	0,6	1,2	1,5	0,5	0,7	6,7

Figure S2: PSCPL matrix for nonameric peptides binding to Acar3. The matrix reflects the peptide-binding properties of Acar3 through the relative binding (RB) value for each amino acid at each position of nonameric peptides from the PSCPL. An RB-value of ≥ 2 defines a favoured amino acid at a specific position, while an RB-value of ≤ 0.5 defines a disfavoured amino acid at a specific position. The anchor-position (AP) value defines the relative contribution of each position to peptide binding (Rasmussen *et al.*, 2014). The logo of the PSCPL-derived Acar3 binding motif ***(Figure 1)*** was made using the Seq2Logo 1.1 (Thomsen & Nielsen, 2012) server as P-Weighted Kullback-Leibler logo.

Figure S3

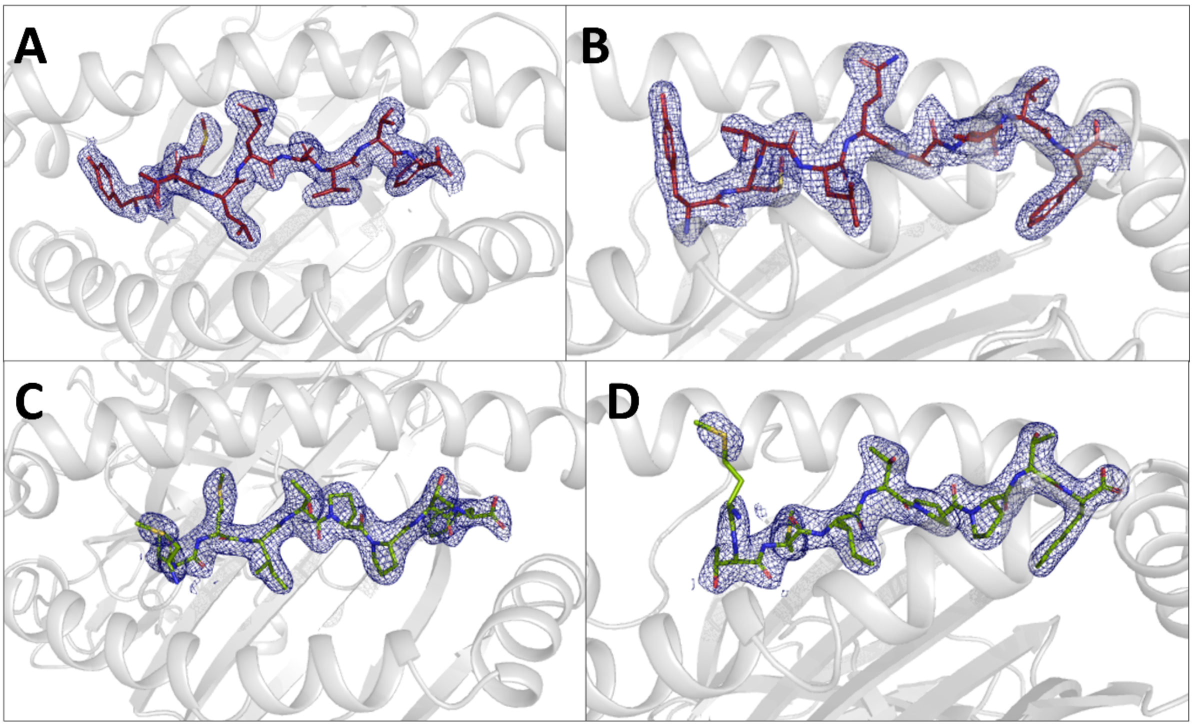

Figure S3: Electron density maps for P2 and P3. Top **(A)** and side **(B)** view of the 2Fo-Fc electron density map of P2 (red, stick representation) shown as blue mesh at a contour level of 1.0 σ. Top **(C)** and side **(D)** view of the 2Fo-Fc electron density map of P3 (green, stick representation) shown as blue mesh at a contour level of 1.0 σ. The Acar3 PBG is shown as cartoon in grey.

Figure S4

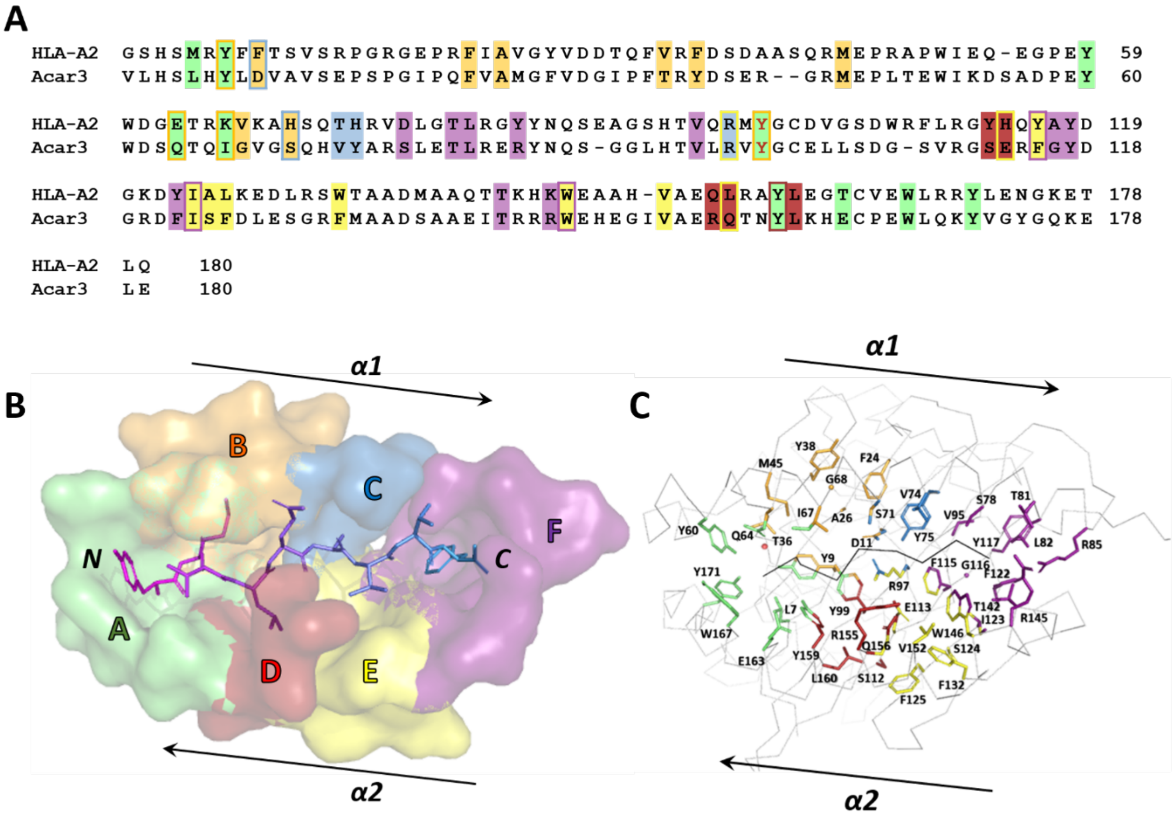

Figure S4: The six pockets of the Peptide Binding Groove (PBG) common among HLA-A2 and Acar3. (A) Alignment of the α1 and α2 domains of the HLA-A2 molecule as described in (Saper *et al.*, 1991) (PDB code 3HLA, aa 1-180) and Acar3 (aa 3-180). The residues that participate in the formation of the six pockets of the PBG are highlighted in different colours. The residues are numbered according to the Acar3 sequence and the HLA-A2 sequence (in parentheses): **Pocket A** (green): L7 (M5), Y9 (Y7), Y60 (Y59), Q64 (E63), I67 (K66), Y99 (Y99), Y159 (Y159), E163 (T163), W167 (W167), and Y171 (Y171); **Pocket B** (orange): Y9 (Y7), D11 (F9), A26 (A24), T36 (V34), M45 (M45), Q64 (E63), I67 (K66), G68 (V67), S71 (H70) and Y99 (Y99). In Acar3, F24 (F22) and Y38 (F36) additionally participate in creating this pocket. **Pocket C** (blue): D11 (F9), S71(H70), V74 (T73), Y75 (H74) and R97 (R97); **Pocket D** (red): Y99 (Y99), the backbone of S112 (Y113); E113 (H114), R155 (Q155), Q156 (L156), Y159 (Y159) and L160 (L160); **Pocket E** (yellow): R97 (R97), E113 (H114), F132 (W133), W146 (W147), V152 (V152) and Q156 (L156). In Acar3, F115 (Y116), I123 (I124) and the backbone atoms of S124 (A125) and F125 (L126) additionally contribute to the pocket surface. **Pocket F** (magenta): S78 (D77), T81 (T80), L82 (L81), R85 (Y84), F115 (Y116), F122 (Y123), T142 (T143), R145 (K146) and W146 (W147). In Acar3, V95 (V95), the backbone atoms of G116 (A117) and Y117 (Y118), and I123 (I124) additionally participate in creating this pocket. Residues that are part of two pockets contain a background and a frame in two different colours according to the colouring scheme of the corresponding pockets. In case a residue belongs to three pockets, the aa letter is coloured corresponding to the third pocket. **(B)** Top view onto the PBG of the Acar3-P2 complex with the pockets as surface representation coloured according to **(A)** and P2 shown in stick representation. **(C)** Stick representation of the pockets' residues coloured according to **(A)**. The Acar3 scaffold and P2 are shown as grey and black ribbon, respectively. **Pocket A** is open towards the solvent and creates an anchor for the N-termini of the bound peptides. A

conserved water molecule (**(C)**, red sphere) is coordinated by Y9, T36, Y60 and Q64 and supports the specific alignment of those residues. The surface potential of the pocket can be altered by the Q64-side chain adapting different orientations. **Pocket B** adopts an elongated shape and has an increased depth and volume compared to pocket B in HLA-A2, which is due to the smaller residues at positions 11, 68 and 71. The pocket hence permits accommodation of bulky residues and harbours the methionine-anchor residue of P2 and P3 (M2 in P2, M3 in P3). Since the M-side chain does not completely fill the cavity, the residual space is occupied by one water molecule in the P3 structure, and two water molecules in the Acar3-P2 complex. The bottom of **pocket C** is formed by the aromatic portion of Y75. S71 and the guanidinium group of R97 create a rim that segregates pocket C from pocket B. According to the shallow and neutral character of pocket C, short and hydrophobic residues, such as A (P2) or P (P3) at position 6 are advantageous for peptide binding. **Pocket D** is separated from the adjacent pocket A by the shared side chain of Y159. This pocket shows different morphologies and surface potentials in the two structures and can likely accommodate amino acids of different shapes and polarities. Residues E113, R155 and Q156 have crucial impact on the shape and surface potential of pocket D and their side-chain conformations vary among both structures. In the Acar3-P2 complex, R155 can be present in two different conformations either directed towards P2 or towards the solvent. **Pocket E** shows some extent of variability between the two structures with surface potential ranging from neutral (P2) to slightly negative (P3). The cavity has a round shape and varies in depth and width depending on the orientation of residues E113, F115, F132, W146 and Q156. Moreover, water molecules can be present in the cavity to mediate hydrogen bonds between the peptide and the PBG residues. **Pocket F** shows a highly positive surface potential around its upper rim as well as clearly positively polarised surface characteristics within the lower parts of the cavity, which is of importance to anchor the C-terminus of the antigenic peptide. The strong polarisation around the rim is primarily achieved through contributions from the guanidinium groups of R85 and R145, as well as W146, whereas the central part and the bottom of the cavity comprise exclusively hydrophobic/aromatic residues.

Figure S5

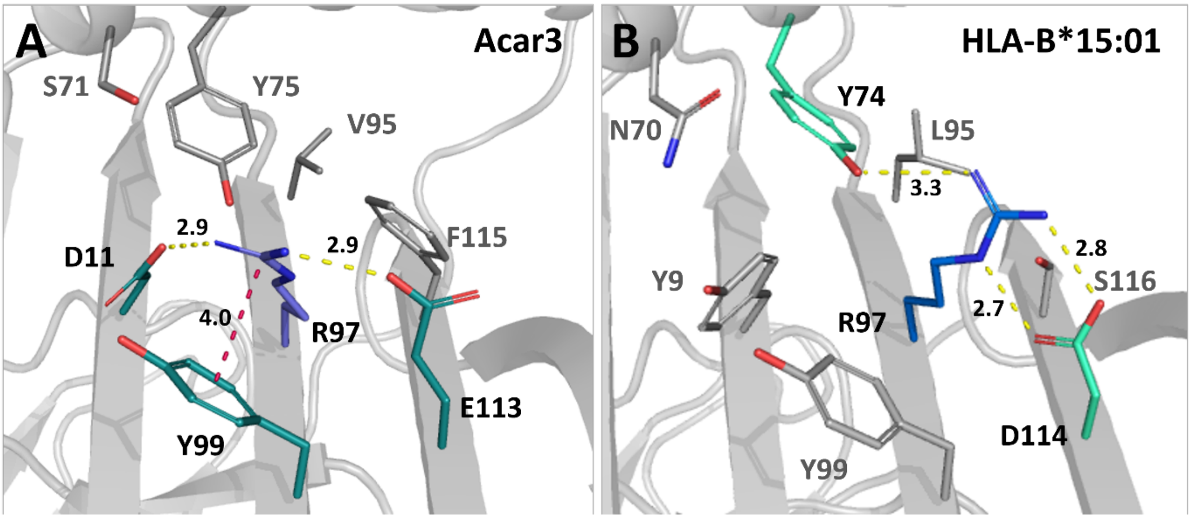

Figure S5: Major interactions of Arg97 in Acar3 and HLA-B*15:01. (A) In Acar3, Arg97 is oriented towards the centre of the PBG, thereby establishing close interactions with Asp11 lining pockets B and C, with Glu113 which is part of pockets D and E, and with Tyr99 flanking pockets A, B and D. **(B)** In HLA-B*15:01, Arg97 is oriented towards the area of the PBG that accommodates the C-terminal residues of bound peptides, forming hydrogen bonds with Tyr74 and Asp114 which belong to pockets C and D/E, respectively. The closest neighbours of Arg97 (blue sticks) in the PBG are shown as sticks; hydrogen-bonding and cation-π interaction partners are highlighted in teal **(A)** and greencyan **(B)**. Hydrogen bonds are indicated as yellow, dashed lines; cation-π interactions are depicted as red, dashed lines. Distances are given in Å.

Figure S6

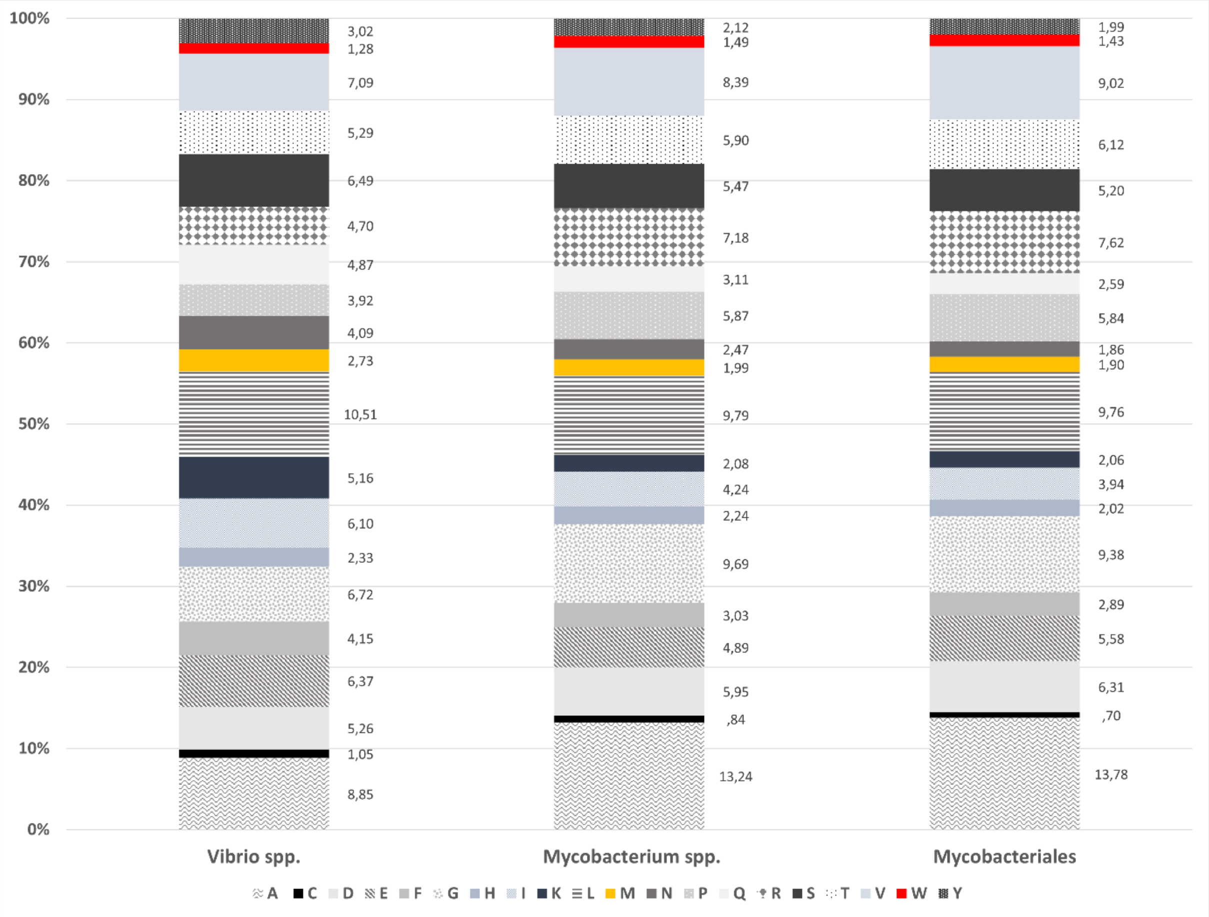

Figure S6: Percentages of amino acids within the proteomes of bacteria representing potential antigen sources for Acar3 binding. Proteomes were retrieved from UniProt (Consortium, 2020). *Vibrio spp.* comprises the proteomes of *V. cholerae* (UP000000584), *V. vulnificus* (UP000002675) and *V. parahaemolyticus* (UP000002493); *Mycobacterium spp.* includes the proteomes of *M. tuberculosis* (UP000001584), *M. paraense* (UP000193285), *M. marinum* (UP000001190), *M. canettii* (UP000008896), *M. simiae* (UP000193040), *M. gordonae* (UP000193928) and *M. malmoense* (UP000243140); and Mycobacteriales contains the proteomes of *Dietzia* and *Tsukamurella spp., i.e. D. cinnamea* (UP000295805), *T. sputi* (UP000319792) and *T. paurometabola* (UP000271626). Amino acids (A-Y) are given in one-letter code.

Table S1: Stability of Acar3 in complex with the peptides obtained through PSCPL analysis

Sequence	Stability T $^1/_2$ (h)	Sequence	Stability T $^1/_2$ (h)
AMSAQAAAF (P1)	11.3	RMMETWHPL	0.9
YMTLQAVTF (P2)	11.2	MMQVWIQPL	0.9
MTMITPPTF (P3)	10.3	FHMSKPSDL	0.8
KTMMQAHDL	9.9	LMMNGTSAM	0.8
QWALLPYLF	9.9	MMWATAQAL	0.8
GMVGAEFSL	7.6	YWMGGTTYF	0.8
RYMSKTYNF	7.5	IYLPIVHPF	0.7
MMMSTAVAF	7.3	GPMKLVMAF	0.7
MMMPMFNAF	7.2	WQLTSIWPI	0.7
RRMATTFTF	6.4	YPMSIPATL	0.7
IFLKPEETF	6.1	FQMGGIGPM	0.7
KAMSTPFSL	5.3	REMINHYQV	0.7
AEMRETHWL	5.0	AVMFFPFWF	0.6
LTMNLVSDI	4.5	QMSWLPLCV	0.6
MMHASTSPF	4.2	LDMNVVKEL	0.6
KQMSQPYAV	4.0	RQNAAIEAL	0.6
AMPKTIYEL	3.8	GAMYALANF	0.6
YQVKYVSPV	3.6	GRLVTVNPF	0.5
MPMSMPIPM	3.2	RMAMIPRTL	0.5
WMMAMRYPI	3.1	VMMAKRPLI	0.5
SPMETTAEF	3.0	YLQQNTHTL	0.5
ETMKPAAMV	2.7	LRWLAKSFF	0.5
YMWECPDFF	2.6	QQMFPGAPF	0.4
LQMLGENVL	2.6	KAMRPWQSF	0.4
KIMQVDRPM	2.6	KMAAFPETL	0.4
AKIEAPLLL	2.5	IRMEFQSVF	0.4
YLYLRPYAL	2.4	KLMDVVYSI	0.4
SLAIDAYPL	2.1	YMWLGARYL	0.4
KMNAKAATL	2.1	LMDENTYAM	0.4
VMPLLVWLF	1.9	QQLEADYTF	0.4
REWSGLNYL	1.9	YLLEMLWRL	0.4
MTYKAAFDL	1.8	KTAAAAAAF	0.4
RRWIAPHPL	1.7	RMMATKDSF	0.4
RMLPKLAEF	1.6	LPLPAQQPF	0.3
SVYFAAFAF	1.6	GMNVTAPAL	0.3
RATQPAAEF	1.6	SQMGLSCAL	0.3
QAYAAPQLF	1.5	VIMWYNYLF	0.3
GLAATSFPL	1.5	LPRWPPPQL	0.3
VPMEKLKAL	1.4	FRMLAWHVL	0.3
IMDASSFTL	1.4	FMIVNNFPV	0.3
KQLESVMYL	1.3	AMAAGLSSL	0.3
FQANMFLTL	1.2	AMMWRIAQL	0.3
FLLENAAYL	1.2	FLDKGTYTL	0.3
HELSLFWPL	1.1	STEFIPNLF	0.3
YMHGSIHEV	0.9	ILMEHIHKL	0.2
SMMPEAMTI	0.9	DYQGLPLEF	0.2
WMACNSAAF	0.9	SYPPPPASF	0.2

Table S2: Interactions of P2 within the Acar3 PBG

P2			Acar3		
Hydrogen bonds (≤ 3.4 Å)					
Residue	*Atom*	*Distance (Å)*	*Residue*	*Atom*	*Pocket*
Y_1	N	2.7	Y171	Oη	A
	N	2.7	Y9	Oη	
	Oη	2.7	S63	Oγ	
	O	2.6	Y159	Oη	
M_2	N	3.3	Q64	Oε1	B
	N	3.2	Y9	Oη	
T_3	Oγ1	3.2	E163	Oε2	-
L_4	O	3.0	R97	Nη1	D
	O	2.8	R97	Nη2	
Q_5	N	3.4	R155	Nη2, A	-
	O	2.3	R155	Nη2, A	
A_6	N	2.8	S71	Oγ	C
V_7	-	-	-	-	E
T_8	Oγ1	3.0	S78	Oγ	-
	O	3.1	W146	Nε1	
F_9	N	3.0	S78	Oγ	F
	O	2.6	T142	Oγ1	
	O	3.2	R145	Nε	
	O	2.8	R85	Nη2	
	OXT	3.3	R145	Nη2	
Water-bridged hydrogen bonds (≤ 3.4 Å)					
Residue (P2)	*Atom*	*Distance (Å)* P2-residue – H_2O	*Distance (Å)* H_2O – Acar3-residue	*Residue (Acar3)*	*Atom*
V_7	N	3.2	2.8	E113	Oε2
			2.8	Q156	Nε2
	O	2.8	2.7	S78	Oγ
T_8	Oγ1	2.7	2.8	T81	Oγ1
F_9	OXT	2.7	2.8	T81	Oγ1
Salt bridges (≤ 4.0 Å)					
Residue	*Atom*	*Distance (Å)*	*Residue*	*Atom*	*Pocket*
F_9	O	3.9	R85	Nη1	F

		2.8		Nη2	
		3.2	R145	Nε	
		3.9		Nη2	
	OXT	4.0	R145	Nε	
		3.3		Nη2	
Hydrophobic contacts/π-stacking (≤ 4.0 Å)					
Residue	*Atom*	*Distance (Å)*	*Residue*	*Atom*	*Pocket*
Y_1	Cβ	4.0	W167	Cβ	A
	Cβ	3.4	W167	Cγ	
	Cβ	3.5	W167	Cδ1	
	Cβ	3.7	W167	Nε1	
	Cβ	3.8	W167	Cε2	
	Cβ	3.6	W167	Cδ2	
	Cγ	4.0	W167	Cδ1	
	Cγ	3.7	W167	Nε1	
	Cγ	3.6	W167	Cε2	
	Cγ	3.9	W167	Cδ2	
	Cγ	4.0	W167	Cζ2	
	Cδ1	3.9	Y60	Cδ1	
	Cδ1	3.6	Y60	Cε1	
	Cδ1	3.7	Q64	Cγ	
	Cδ1	3.7	Q64	Cδ	
	Cε1	4.0	Y60	Cδ1	
	Cε1	4.0	Y60	Cδ1	
	Cε1	3.7	Q64	Cγ	
	Cε1	4.0	Q64	Cδ	
	Cε2	3.8	W167	Nε1	
	Cε2	4.0	W167	Cε2	
	Cε2	3.8	W167	Cζ2	
	Cδ2	3.7	W167	Cδ1	
	Cδ2	3.1	W167	Nε1	
	Cδ2	3.4	W167	Cε2	
	Cδ2	3.7	W167	Cζ2	
	C	3.9	Y9	Cζ	
	C	4.0	Y9	Cε2	
M_2	Cα	3.8	Y9	Cζ	B
	Cβ	3.7	Y9	Cε1	
	Cβ	3.8	Y9	Cζ	
	Cγ	4.0	Y9	Cε1	
	Cγ	4.0	Y9	Cδ1	
	Sδ	3.6	A26	Cβ	
	Sδ	4.0	M45	Sδ	
	Sδ	3.7	M45	Cε	
	Cε	3.9	M45	Sδ	
	Cε	3.5	G68	Cα	
T_3	Cα	3.9	I67	Cγ1	-
	Cα	4.0	I67	Cδ1	
L_4	Cβ	3.9	Y99	Cε2	D

	Cβ	3.8	Y159	Cγ	
	Cβ	4.0	Y159	Cδ1	
	Cβ	4.0	Y159	Cδ2	
	Cδ1	3.7	Y159	Cβ	
	Cδ1	3.9	Y159	Cγ	
	Cδ1	3.8	R155	Cζ, A	
	Cδ1	3.8	R155	Cγ, A	
	Cδ2	3.8	Y159	Cδ2	
	Cδ2	3.9	Y99	Cδ2	
Q_5	Cα	3.6	I67	Cγ2	-
	Cβ	3.8	I67	Cγ2	
	Cγ	4.0	G70	C	
	Cδ	3.8	G70	Cα	
	Cδ	3.9	G70	C	
A_6	Cα	3.9	R97	Cζ	C
	Cβ	3.9	Y75	Cε1	
	Cβ	3.5	Y75	Cζ	
	Cβ	3.9	Y75	Cε2	
	Cβ	3.8	V74	Cγ1	
	C	3.7	V74	Cγ1	
V_7	Cβ	3.9	W146	Cζ2	E
	Cγ1	3.7	W146	Cε2	
	Cγ1	3.9	W146	Cζ2	
	C	3.6	V74	Cγ1	
	C	4.0	W146	Cζ2	
T_8	Cα	3.6	V74	Cγ1	-
	Cβ	3.9	V74	Cγ1	
F_9	Cγ	3.8	F122	Cε2	F
	Cδ1	3.8	W146	Cζ2	
	Cδ1	3.7	F122	Cε2	
	Cε1	3.9	F115	Cγ	
	Cε1	3.6	F115	Cδ2	
	Cε1	3.8	F115	Cε2	
	Cε1	4.0	F122	Cδ2	
	Cε1	3.9	F122	Cε2	
	Cζ	3.8	V95	Cγ1	
	Cζ	3.9	F115	Cγ	
	Cζ	3.4	F115	Cδ2	
	Cζ	3.9	F115	Cε2	
	Cζ	3.9	F122	Cδ2	
	Cδ2	3.7	S78	Cβ	
	Cδ2	3.8	L82	Cγ	
	Cδ2	3.6	L82	Cδ1	
	Cδ2	3.9	F122	Cε2	
	Cε2	3.8	V95	Cγ1	
	Cε2	3.8	L82	Cδ1	
	Cε2	4.0	F122	Cε2	

Table S3: Interactions of P3 within the Acar3 PBG

P3			Acar3		
Hydrogen bonds (≤ 3.4 Å)					
Residue	*Atom*	*Distance (Å)*	*Residue*	*Atom*	*Pocket*
M_1	O	3.2	Q64	Nε2	-
T_2	Oγ1	2.7	Y9	Oη	A
	Oγ1	2.5	Y171	Oη	
	O	2.5	Y159	Oη	
M_3	-	-	-	-	B
I_4	N	3.1	Y99	Oη	D
	O	3.0	R97	Nη1	
	O	3.2	R97	Nη2	
T_5	Oγ	2.4	R155	Nη2	-
	O	2.8	R155	Nη1	
	O	3.2	R155	Nη2	
P_6	-	-	-	-	C
P_7	-	-	-	-	E
T_8	O	3.0	R145	Nη2	-
	O	3.1	W146	Nε1	
F_9	N	3.3	S78	Oγ	F
	O	3.3	R85	Nη2	
	O	3.0	T142	Oγ1	
	O	2.6	R145	Nη1	
Water-bridged hydrogen bonds (≤ 3.4 Å)					
Residue (P3)	*Atom*	*Distance (Å)* P2-residue – H_2O	*Distance (Å)* H_2O – Acar3-residue	*Residue (Acar3)*	*Atom*
P_7	O	2.8	2.6	S78	Oγ
Salt bridges (≤ 4.0 Å)					
Residue	*Atom*	*Distance (Å)*	*Residue*	*Atom*	*Pocket*
F_9	O	3.3	R85	Nη2	F
		2.6	R145	Nη1	
		3.6		Nη2	
Hydrophobic contacts/π-stacking (≤ 4.0 Å)					
Residue	*Atom*	*Distance (Å)*	*Residue*	*Atom*	*Pocket*
M_1	Cβ	4.0	I67	Cδ1	-

	Cγ	4.0	I67	Cδ1	
T_2	Cα	3.8	W167	Cδ1	A
	Cα	4.0	W167	Cγ	
	Cβ	3.7	W167	Cγ	
	Cβ	4.0	W167	Cδ1	
	Cβ	3.9	W167	Cδ2	
	Cγ2	3.2	Q64	Cδ	
	Cγ2	3.9	Y60	Cε1	
	C	3.9	Y9	Cε2	
	C	3.8	Y159	Cε1	
M_3	Cβ	3.8	I67	Cγ2	B
	Cγ	3.9	Y9	Cγ	
	Cγ	3.5	Y9	Cδ1	
	Cγ	3.5	Y9	Cε1	
	Cγ	3.9	Y9	Cζ	
	Sδ	4.0	Y9	Cδ1	
	Sδ	4.0	Y9	Cε1	
	Sδ	3.7	M45	Cε	
	Sδ	3.9	A26	Cβ	
	Cε	3.5	I67	Cγ2	
I_4	Cγ1	3.7	Y99	Cε2	D
	Cγ1	4.0	Y159	Cε2	
	Cγ1	3.8	Y159	Cγ	
	Cγ1	3.9	Y159	Cδ2	
	Cγ1	4.0	Y159	Cε1	
	Cγ1	4.0	Y159	Cζ	
	Cγ1	3.9	Y159	Cδ1	
	Cγ2	3.9	Y159	Cγ	
	Cγ2	3.5	Y159	Cδ1	
	Cδ1	4.0	Y159	Cε2	
	Cδ1	3.8	Y159	Cβ	
	Cδ1	3.5	Y159	Cγ	
	Cδ1	4.0	Y159	Cδ1	
	Cδ1	3.4	Y159	Cδ2	
	C	4.0	I67	Cγ2	
T_5	-	-	-	-	-
P_6	Cα	3.6	R97	Cζ	C
	Cβ	4.0	F115	Cε2	
	Cβ	3.9	Y75	Cε1	
	Cβ	3.8	Y75	Cζ	
	Cβ	3.8	R97	Cζ	
	Cγ	4.0	V74	Cβ	
	Cγ	3.8	V74	Cγ1	
	Cγ	3.8	Y75	Cε1	
	Cγ	3.6	Y75	Cζ	
	Cγ	3.8	Y75	Cε2	
	Cδ	4.0	S71	Cα	

	Cδ	3.7	S71	Cβ	
P_7	Cβ	3.9	W146	Cε2	E
	Cβ	3.6	V152	Cγ1	
	Cβ	3.6	W146	Cζ2	
	Cγ	4.0	W146	Cζ2	
	Cγ	3.5	V152	Cγ1	
	Cγ	4.0	Q156	Cδ	
	Cδ	3.6	F115	Cζ	
T_8	Cα	3.8	V74	Cγ1	-
	Cβ	4.0	V74	Cγ1	
F_9	Cγ	3.9	F122	Cε2	F
	Cδ1	3.9	F112	Cε2	
	Cδ1	4.0	T142	Cγ2	
	Cδ1	3.9	W146	Cζ2	
	Cε1	4.0	F122	Cδ2	
	Cε1	3.9	F122	Cε2	
	Cε1	4.0	W146	Cη2	
	Cε1	3.9	F115	Cδ2	
	Cε1	4.0	F115	Cε2	
	Cζ	3.7	F122	Cδ2	
	Cζ	3.9	F122	Cε2	
	Cζ	4.0	F115	Cβ	
	Cζ	3.7	F115	Cγ	
	Cζ	3.4	F115	Cδ2	
	Cζ	3.9	F115	Cε2	
	Cδ2	3.8	L82	Cγ	
	Cδ2	3.7	L82	Cδ1	
	Cδ2	3.8	L82	Cδ2	
	Cδ2	3.8	F122	Cε2	
	Cδ2	3.6	S78	Cβ	
	Cε2	3.8	L82	Cδ1	
	Cε2	3.8	F122	Cδ2	
	Cε2	3.8	F122	Cε2	
	C	4.0	R145	Cζ	

Table S4: Comparison of r.m.s.d. values of anchor residues and terminal and central segments of peptides bound to Acar3, HLA-A*24:02 and HLA-B*15:01.

			r.m.s.d. range/average (Å)* of:				
**PDB code**	**Anchor residues/ positions**	**Peptide length (# of aa)**	**N-terminal anchor**	**aa 1-3****	**aa 4-6****	**aa 7-9****	**C-terminal anchor**
					Acar3		
7ZQI (P2)	M/2, F/9	9	- /**0.87**	0.87-1.31 /**1.09**	0.64-1.60 /**1.01**	0.96-1.22 /**1.07**	- /**1.03**
7ZQJ (P3)	M/3, F/9	9					
					HLA-A*24:02		
2BCK (A, B, C)	Y/2, L/9	9	0.15-0.58 /**0.35**	0.48-0.54 /**0.52**	1.29-3.16 /**2.27**	0.87-1.32 /**1.10**	0.11-0.95 /**0.66**
3I6L	F/2, L/9	9					
4F7M (A, B, C)	Y/2, F/10	10					
4F7T (A, B, C)	Y/2, F/8	8					
5WWU	Y/2, F/11	11					
5WXD	Y/2, F/11	11					
					HLA-B*15:01		
1XR8	E/2, Y/9	9	0.02-0.72 /**0.48**	0.02-1.48 /**0.50**	0.45-3.57 /**1.75**	0.12-1.29 /**0.52**	0.12-1.16 /**0.59**
1XR9	L/2, Y/9	9					
3C9N	Q/2, M/9	9					
5TXS	Q/2, Y/9	9					
5VZ5	Q/2, Y/10	10					

* The values were obtained using *Superpose* (Krissinel & Henrick, 2004) (*gesamt* method) from the *CCP4i program suite* (version 7.0.078) (Winn *et al.*, 2011) calculating r.m.s.d. values for all possible combinations of structures from the same protein. In HLA-A*24:02, conformational changes of F99 and Y159 may induce an expansion of pocket B. However, this mechanism rather enables the accommodation of a secondary anchor located at peptide position 3 [11] than providing additional flexibility to the primary anchor only. Consequently, those structures have been excluded from the r.m.s.d. calculations.

** The values each comprise the r.m.s.d. range and average of the three N-terminal, central and C-terminal residues combined from all structures containing nonameric peptides in all possible combinations. Due to the N-terminal variations of the peptides in the Acar3 structures, the superposition of P2 *vs.* P3 only contains two overlaps, *i.e.* residue 1 (P2) with residue 2 (P3) and residue 2 (P2) with residue 3 (P3).

Table S5: Half-life for peptide-MHC-I molecules for a series of peptide - Acar3 and - Acar19 combinations estimated using the dissociation using a scintillation proximity assay.

Sequence	Stability T½ (h)	Acar
AANMYIYPL	0.1	Acar3
FQFTLHWEL	0.0	Acar3
KEISNQEPL	0.0	Acar3
QQYAGWSAL	0.0	Acar3
FLQGAKWYL	0.1	Acar3
IMDASSFTL	1.4	Acar3
KLMDVVYSI	0.4	Acar3
SLAIDAYPL	2.1	Acar3
AQRELFFTL	0.0	Acar3
YLLADTFTV	0.0	Acar3
VQYRGLYQI	0.0	Acar3
RLHDAWWTL	0.0	Acar3
SLAALFYSL	0.0	Acar3
AMMWRIAQL	0.3	Acar3
AELGAFFSI	0.0	Acar3
MMHASTSPF	4.2	Acar3
YLAENTFVV	0.0	Acar3
KQLEWKWGI	0.0	Acar3
WLAKSFFEL	0.2	Acar3
SQEDNHFSL	0.0	Acar3
AQIGIFAPV	0.0	Acar3
KEMGFSPRL	0.0	Acar3
SESTHYFTV	0.0	Acar3
GLAATSFPL	1.5	Acar3
GQWGGDWAV	0.0	Acar3
SQDDNYFTL	0.0	Acar3
LEMQHLISL	0.0	Acar3
FQMGGIGPM	0.7	Acar3
AQMVWIHGV	0.0	Acar3

YMHGSIHEV	0.9	Acar3
KQMEDGHTL	0.0	Acar3
AEMRETHWL	5.0	Acar3
SQAAFGLPI	0.0	Acar3
KQLELFWVI	0.2	Acar3
WQTDTTIPL	0.0	Acar3
FLLENAAYL	1.2	Acar3
WQLTSIWPI	0.7	Acar3
NQFGSVPAL	0.0	Acar3
AEAASATPL	0.0	Acar3
FMIVNNFPV	0.3	Acar3
RQWGMGFLL	0.0	Acar3
LQAMHGFPL	0.0	Acar3
MMQVWIQPL	0.9	Acar3
TLKRRSWPL	0.0	Acar3
WMACNSAAF	0.9	Acar3
KQSSFLSSL	0.0	Acar3
YEFLGELAL	0.0	Acar3
YLQQNTHTL	0.5	Acar3
RQNAAIEAL	0.6	Acar3
SQAFNTPAL	0.0	Acar3
AELRHLNPL	0.0	Acar3
YLACKQHAL	0.0	Acar3
YQAGISAAL	0.0	Acar3
SMFERDFHF	0.2	Acar3
RLMKQDFSV	0.0	Acar3
YLYLRPYAL	2.4	Acar3
FQANMFLTL	1.2	Acar3
REWSGLNYL	1.9	Acar3
YQVKYVSPV	3.6	Acar3
FQWWRSHPL	0.0	Acar3
LEMCHSTQI	0.0	Acar3

FLDKGTYTL	0.3	Acar3
REAGMAATL	0.0	Acar3
FMYSDFHFI	0.1	Acar3
YLMPYSVYI	0.1	Acar3
KQLESVMYL	1.3	Acar3
ILMEHIHKL	0.2	Acar3
KQWSWFSLL	0.0	Acar3
LMDENTYAM	0.4	Acar3
RQLLWRYQI	0.0	Acar3
HELSLFWPL	1.1	Acar3
MQQGRFPPL	0.0	Acar3
RQQAIVDLL	0.0	Acar3
RENGGYWLL	0.0	Acar3
FMFDYIPPV	0.2	Acar3
YQGSYGFRL	0.0	Acar3
KQLEYSWVL	0.1	Acar3
SQGRGWFLL	0.0	Acar3
SMHFYGWSL	0.2	Acar3
FQAQNIAGL	0.0	Acar3
RMFKRVFNM	0.0	Acar3
RQIRMTSTI	0.1	Acar3
KAYANMWSL	0.0	Acar3
RQYERYTAL	0.0	Acar3
MQLPGGWLL	0.0	Acar3
YQLGDYFFV	0.0	Acar3
WEFVNRPPL	0.0	Acar3
SQIETGTPF	0.0	Acar3
AMPKTIYEL	3.8	Acar3
YLLEMLWRL	0.4	Acar3
SQMGLSCAL	0.3	Acar3
FQNWGIESI	0.0	Acar3
REMINHYQV	0.7	Acar3

AMAAGLSSL	0.3	Acar3
DMSTNAEYF	0.0	Acar3
GRWMLPQGM	0.0	Acar3
KRKKAYADF	0.0	Acar3
QAYAAPQLF	1.5	Acar3
VQTAAAVVF	0.2	Acar3
VMMAKRPLI	0.5	Acar3
NTDEIPELI	0.0	Acar3
IYLPIVHPF	0.7	Acar3
GAMYALANF	0.6	Acar3
YMKKRYEEF	0.2	Acar3
AKIEAPLLL	2.5	Acar3
WMMAMRYPI	3.1	Acar3
KMNAKAATL	2.1	Acar3
WRTIMAVLF	0.0	Acar3
ERWFVRNPF	0.0	Acar3
MTMITPPTF	10.3	Acar3
QQMFPGAPF	0.4	Acar3
AVMFFPFWF	0.6	Acar3
TRAPAPFPL	0.1	Acar3
GFMNEDHWF	0.0	Acar3
DYQGLPLEF	0.2	Acar3
EEMPLVWDL	0.0	Acar3
KTAAAAAAF	0.4	Acar3
YMWLGARYL	0.4	Acar3
GMNVTAPAL	0.3	Acar3
GRLVTVNPF	0.5	Acar3
LDMNVVKEL	0.6	Acar3
VTTPARTAF	0.0	Acar3
MMMPMFNAF	7.2	Acar3
YMTLQAVTF	11.2	Acar3
VIMWYNYLF	0.3	Acar3

SVYFAAFAF	1.6	Acar3
ETTQALQLF	0.0	Acar3
MPMSMPIPM	3.2	Acar3
FRMLAWHVL	0.3	Acar3
RRMATTFTF	6.4	Acar3
QMSWLPLCV	0.6	Acar3
LRQWAPATM	0.0	Acar3
KAMRPWQSF	0.4	Acar3
GTVPTDNPF	0.0	Acar3
LMMNGTSAM	0.8	Acar3
QQLEADYTF	0.4	Acar3
MTYKAAFDL	1.8	Acar3
YYVWMVQFF	0.0	Acar3
DIIRAHPWF	0.0	Acar3
LPRWPPPQL	0.3	Acar3
RMMETWHPL	0.9	Acar3
FHMSKPSDL	0.8	Acar3
LRWLAKSFF	0.5	Acar3
KAMSTPFSL	5.3	Acar3
LTMNLVSDI	4.5	Acar3
KIMQVDRPM	2.6	Acar3
SYPPPPASF	0.2	Acar3
DTDPLPVVF	0.0	Acar3
VTDTALAYF	0.0	Acar3
QPWTPVSSF	0.0	Acar3
VPMEKLKAL	1.4	Acar3
LQMLGENVL	2.6	Acar3
GMVGAEFSL	7.6	Acar3
IRMEFQSVF	0.4	Acar3
RMAMIPRTL	0.5	Acar3
RMMATKDSF	0.4	Acar3
RMLPKLAEF	1.6	Acar3

VVKQLPASF	0.1	Acar3
RMMGKTNPL	0.2	Acar3
STEFIPNLF	0.3	Acar3
VMPLLVWLF	1.9	Acar3
SPMETTAEF	3.0	Acar3
NMKWKFNAL	0.0	Acar3
YMWECPDFF	2.6	Acar3
LPLPAQQPF	0.3	Acar3
KRLLLKLDF	0.0	Acar3
WMYNIQPYL	0.0	Acar3
RATQPAAEF	1.6	Acar3
NMAPEKVDF	0.0	Acar3
KMAAFPETL	0.4	Acar3
ETMKPAAMV	2.7	Acar3
YWMGGTTYF	0.8	Acar3
RYMSKTYNF	7.5	Acar3
GPMKLVMAF	0.7	Acar3
EFKQILTDF	0.0	Acar3
RRWIAPHPL	1.7	Acar3
MMMSTAVAF	7.3	Acar3
KRMMVRHCL	0.0	Acar3
MMWATAQAL	0.8	Acar3
KTMMQAHDL	9.9	Acar3
AMSAQAAAF	11.3	Acar3
SMMPEAMTI	0.9	Acar3
KQMSQPYAV	4.0	Acar3
IFLKPEETF	6.1	Acar3
VWKQLFPEL	0.0	Acar3
YPMSIPATL	0.7	Acar3
QWALLPYLF	9.9	Acar3
DMWEHAFYL	0.0	Acar3
DAAKNQVAM	0.2	Acar19

TFMYVFSTF	0.2	Acar19
NTDEIPELI	0.2	Acar19
NTFAAWLPM	0.6	Acar19
DPNPQEVVL	0.4	Acar19
QYQYLSILF	0.0	Acar19
LYGLITEQF	0.0	Acar19
TYSPALNKM	0.1	Acar19
MYLRTIYDL	4.0	Acar19
QYPAFVLFI	0.2	Acar19
ESFDLAGLF	0.0	Acar19
VMKRNFIDF	0.1	Acar19
IYASRPLDF	0.0	Acar19
EYARQVQMI	0.4	Acar19
MSADNAGAL	0.0	Acar19
VSFSMVGLF	0.1	Acar19
NPSVLKILL	0.2	Acar19
TRAPAPFPL	0.1	Acar19
DFIGKTIGF	0.0	Acar19
YQAGISAAL	0.2	Acar19
TYMFTHIDL	0.1	Acar19
GVPELGAFF	0.0	Acar19
SFHIIEWLF	0.2	Acar19
NAFHHPSAV	0.1	Acar19
SYLGLTQPF	0.2	Acar19
SQAPLPCVL	0.0	Acar19
IYPGVNHGF	0.0	Acar19
DISPTNIPL	0.2	Acar19
EGAGIDDPV	0.2	Acar19
YPMSIPATL	0.1	Acar19
GAAGARGAL	0.0	Acar19
IASPAWFLF	0.3	Acar19
ITAALAWSL	0.1	Acar19

IYGIFQSTF	0.1	Acar19
LANYAFFAI	0.0	Acar19
DPKKTGGPI	0.0	Acar19
AYARNLDTL	0.0	Acar19
DFIANTETI	4.7	Acar19
DPLVIPFSF	0.0	Acar19
RYFTVAFLF	0.0	Acar19
GHFPLQHAL	0.2	Acar19
HTAEIQQFF	0.3	Acar19
IAARAVELL	0.0	Acar19
RFNNLTVYF	0.0	Acar19
ISASLAALF	0.1	Acar19
DVVPMVTQM	0.6	Acar19
YWARATVEL	0.0	Acar19
RYSGFVRTL	0.0	Acar19
EFVSANLAM	0.0	Acar19
EDFEIFYNL	0.4	Acar19
YTYPIAHTA	0.1	Acar19
RYAYTSVEF	0.0	Acar19
WDAYIPHYV	0.0	Acar19
ITFHNQRDF	0.2	Acar19
KYNSHHFTF	0.0	Acar19
YISPIFIPM	0.1	Acar19
DSPATLSAY	0.0	Acar19
RYPGVMYAF	0.0	Acar19
ITLPISASL	0.0	Acar19
DYLVSTQEF	0.0	Acar19
AFHQLVQVI	0.3	Acar19
ITDWLNFTL	0.1	Acar19
MYLRFAERL	0.2	Acar19
IAKGIDAEF	0.1	Acar19
DHIPIINTL	0.3	Acar19

DMNLEQWSV	0.4	Acar19
QFAGGSFDF	0.0	Acar19
HAATNFREI	0.1	Acar19
DAAVVFPPV	10.0	Acar19
ATPYNPEDI	0.0	Acar19
EFKQILTDF	3.6	Acar19
LYIEILRLL	0.1	Acar19
VFSPFGYSF	0.0	Acar19
IFPSMYFLM	0.1	Acar19
IQNATMDEF	0.1	Acar19
KYAKFFQNF	0.0	Acar19
FSIMIYFTF	0.2	Acar19
DTRAIDQFF	2.3	Acar19
IALPVAWLF	0.1	Acar19
TTLSIYFLL	0.1	Acar19
LRHSTAHLL	0.1	Acar19
KASSAWHYF	0.2	Acar19
NYNGLLSSI	19.1	Acar19
WYAPVALLF	0.2	Acar19
RYPAIAGGI	0.0	Acar19
LFPELDCFF	1.6	Acar19
DSIMLTATF	1.5	Acar19
LMNELGVPF	0.6	Acar19
DTFGVIDTM	0.2	Acar19
WYIKIFIII	0.0	Acar19
FFKCIYRLF	0.0	Acar19
DVKFHTQAF	0.0	Acar19
IQNALEKAL	0.1	Acar19
YSRMLYIEF	0.0	Acar19
YTIDLNDAF	0.0	Acar19
NMAPEKVDF	0.2	Acar19
RYSNFAWYF	0.0	Acar19

ISPRNYFTF	0.0	Acar19
YYNAFQWAI	0.4	Acar19
YQAYAAPQL	0.0	Acar19
NPLEIYQEI	0.8	Acar19
DVSPILEAY	0.1	Acar19
KSNGAQQWL	0.1	Acar19
RYWYFAAEL	0.0	Acar19
DGFGVHLAF	0.4	Acar19
NVGRILGYV	0.5	Acar19
DALLNEENL	2.0	Acar19
HYNAFHWAI	0.3	Acar19
DQIRNMSVI	0.3	Acar19
KSAQVPLPL	0.0	Acar19
KDPENFDHF	0.2	Acar19
LSDAIFDDL	0.0	Acar19
NTARLMAGA	0.2	Acar19
IPKYLEIEI	0.2	Acar19
IAASNLEQF	0.1	Acar19
NFGRVKVHF	0.6	Acar19
DYQGLPLEF	3.5	Acar19
EAAKNVECI	0.4	Acar19
IASPILFPF	0.5	Acar19
IPAPGLGAL	0.2	Acar19
YYSNKVFPI	0.2	Acar19
YRSMIAFKL	0.1	Acar19
YYNAFHWAI	0.4	Acar19
ENALLVALF	0.1	Acar19
QRASNVFDL	0.2	Acar19
EYAPFARLL	1.1	Acar19
VYFVLTDRF	0.0	Acar19
MAAAAFPAL	0.0	Acar19
DWMERIEDF	1.4	Acar19

FFSPVIASL	0.9	Acar19
HYANFHNYF	0.0	Acar19
VYDPLQPEL	0.0	Acar19
ISDPLTSGL	0.1	Acar19
MFMMIFHFV	0.4	Acar19
ISARGQELF	0.1	Acar19
DTETLTTMF	0.1	Acar19
YYKKTFSAL	0.0	Acar19
EYKKFIATF	0.0	Acar19
SYLQAIGIL	0.1	Acar19
IQNKLSSTF	0.2	Acar19
MPARLWLCL	0.9	Acar19
IAAPNMIAV	0.1	Acar19
VSARIAALF	0.0	Acar19
KFHQIEKEF	0.3	Acar19
MYYPAQLYL	0.0	Acar19
DYMPSMKRF	0.0	Acar19
MFGYNPYSL	0.0	Acar19
HYNAFQWAI	0.3	Acar19
LAASVFFTF	0.2	Acar19
FWPQHFGLI	0.1	Acar19
ITNPFFYQM	0.0	Acar19
EYLRIPLTI	0.0	Acar19
MYPFIFFIV	0.0	Acar19
DFISMYFPW	0.5	Acar19
YFSGIMVRL	0.4	Acar19
DMWEHAFYL	0.3	Acar19
KYAEAFQMV	0.3	Acar19
RMAMIPRTL	0.0	Acar19
NYPASLHKF	0.3	Acar19
DSFPNSVTI	1.4	Acar19
DVAKIEAPL	11.4	Acar19

YTMELCGAM	0.1	Acar19
GFNKLRSTL	0.4	Acar19
VTNRHEEKF	0.1	Acar19
LFQPLHTVM	0.0	Acar19
SHAAIGAYL	0.0	Acar19
ISGPIKHPL	0.0	Acar19
STAPTGSWF	0.2	Acar19
EAPENAREL	0.0	Acar19
DAMENPLSL	0.2	Acar19
KSAQFPFHF	0.0	Acar19
GYAGTLQSL	0.0	Acar19
IFDDLQGSL	0.1	Acar19
RPAPATGAL	0.3	Acar19
GFPSLESSF	0.1	Acar19
QWALLPYLF	0.0	Acar19
SYSGQRTEL	0.0	Acar19
YFDPANGKF	0.1	Acar19
IPNYLEIEI	0.6	Acar19
TFMGMNVQF	0.1	Acar19
ITPTIYLLL	0.0	Acar19
RYMYIYLFI	0.0	Acar19
AYAGLFTPL	0.4	Acar19
DYKECEWPL	17.5	Acar19
QYSGFVRTL	0.0	Acar19
IYNTVVLTI	0.0	Acar19
SFIPIFYQF	1.2	Acar19
GYAWIDFDI	0.2	Acar19
IYKNKISDF	0.0	Acar19
IFFFLFNIL	0.0	Acar19
DVSPLMHLF	2.9	Acar19
YFNTHDVYF	0.0	Acar19
DFGYATMAK	0.3	Acar19

DAVRNAAAI	0.3	Acar19
SYAAAQRKL	0.0	Acar19
IYYWTAWLI	0.2	Acar19
NNKSRLVAF	0.0	Acar19
RMPPLGHEL	0.0	Acar19
FSPRLCRPF	0.4	Acar19
NHIRIEYII	0.4	Acar19
AYPELACAV	0.0	Acar19
IYLRRYFEI	0.0	Acar19
RYVGLYLPF	0.0	Acar19
IYWLIFWRF	0.0	Acar19
SYQALAFDI	0.5	Acar19
ETFRLLRSF	0.1	Acar19
FYPPVLQPI	0.3	Acar19
FFSPFFFSL	0.2	Acar19
LANPTADDF	0.0	Acar19
VYAPAGVEL	0.0	Acar19
WFGHLASDW	0.1	Acar19
IYLPIVHPF	0.0	Acar19
RSFRIHILF	0.0	Acar19
TTKPFWFQL	0.0	Acar19
SSNPVMSRF	0.1	Acar19
TYLALMATF	0.1	Acar19
NYFNRMFHF	0.7	Acar19
YYYNFSEDL	0.0	Acar19
QTSSIEGAW	0.1	Acar19
MGAGLVFPI	0.0	Acar19
DYAMHGTVF	2.4	Acar19
LYPTFYCLF	0.4	Acar19
STYGISEDL	0.1	Acar19
DVSEQLELI	0.0	Acar19
NRYGLPEKM	0.0	Acar19

DIQKLVGKL	0.4	Acar19
ENARLRALL	0.0	Acar19
DASFNASAI	1.0	Acar19
DMSTNAEYF	0.2	Acar19
DYLTTYFTW	0.0	Acar19
NFWLNTLLF	3.4	Acar19
MYMALIAAF	0.3	Acar19
DFNEAIQAY	0.3	Acar19
MYQYIFLSF	0.0	Acar19
MAMGILHTI	0.1	Acar19
LSNFMLWQF	0.0	Acar19
LYNTAIVGL	0.0	Acar19
QFLSFASLF	0.0	Acar19
IYAAISYMI	0.3	Acar19
RFNAIWFNH	0.0	Acar19
RAAPLMQSL	0.0	Acar19
MSNPLTSPI	6.3	Acar19
VQHNIKHSF	0.1	Acar19
EWAENCYNL	0.0	Acar19
ISKKAKGWF	0.0	Acar19
TATPAWDAL	0.1	Acar19
TVADIWHAM	0.1	Acar19
NYMPYVFTL	14.9	Acar19
AFSPNRFWM	0.0	Acar19
DPSGAYFAW	0.1	Acar19
DWSGYSGSF	0.3	Acar19
STFAASGPF	0.0	Acar19
YNAELLVAL	0.2	Acar19
DYLQYVLQI	0.5	Acar19
HYQTLCTNF	0.0	Acar19
IYTVIYYIF	0.2	Acar19
TTNGCSQAM	0.0	Acar19

ITGQIIFGF	0.0	Acar19
ISPGNKSDM	0.0	Acar19
IANTTDHFF	0.2	Acar19
TNTQNNDWF	0.2	Acar19
AFHGLDVKF	0.0	Acar19
IQNEIMKPL	0.2	Acar19
DTAKAFAYL	0.5	Acar19
YVFAIPLPF	0.0	Acar19
DYLQCVLQI	6.6	Acar19
DIKLIDIAL	3.7	Acar19
DPNPQEMVL	0.1	Acar19
ISSGCTKTF	0.1	Acar19
ESPINQIAF	0.0	Acar19
DTDPLPVVF	0.2	Acar19
DYDDVVHEV	0.2	Acar19
ITPKTHFLF	0.0	Acar19
PYPYLFYKF	0.0	Acar19
EAAGIAACL	0.6	Acar19

Consortium, T. U. (2020). UniProt: the universal protein knowledgebase in 2021. *Nucleic acids research, 49*(D1), D480-D489. doi:10.1093/nar/gkaa1100

Krissinel, E., & Henrick, K. (2004). Secondary-structure matching (SSM), a new tool for fast protein structure alignment in three dimensions. *Acta Crystallogr D Biol Crystallogr, 60*(Pt 12 Pt 1), 2256-2268. doi:10.1107/s0907444904026460

Rasmussen, M., Harndahl, M., Stryhn, A., Boucherma, R., Nielsen, L. L., Lemonnier, F. A., . . . Buus, S. (2014). Uncovering the peptide-binding specificities of HLA-C: a general strategy to determine the specificity of any MHC class I molecule. *J Immunol, 193*(10), 4790-4802. doi:10.4049/jimmunol.1401689

Saper, M. A., Bjorkman, P. J., & Wiley, D. C. (1991). Refined structure of the human histocompatibility antigen HLA-A2 at 2.6 Å resolution. *Journal of Molecular Biology, 219*(2), 277-319. doi:https://doi.org/10.1016/0022-2836(91)90567-P

Thomsen, M. C. F., & Nielsen, M. (2012). Seq2Logo: a method for construction and visualization of amino acid binding motifs and sequence profiles including sequence weighting, pseudo counts and two-sided representation of amino acid enrichment and depletion. *Nucleic acids research, 40*(Web Server issue), W281-W287. doi:10.1093/nar/gks469

Winn, M. D., Ballard, C. C., Cowtan, K. D., Dodson, E. J., Emsley, P., Evans, P. R., . . . Wilson, K. S. (2011). Overview of the CCP4 suite and current developments. *Acta Crystallogr D Biol Crystallogr, 67*(Pt 4), 235-242. doi:10.1107/s0907444910045749
